# Chronotype at the beginning of secondary school and school timing are both associated with chronotype development during adolescence

**DOI:** 10.1038/s41598-022-11928-9

**Published:** 2022-05-17

**Authors:** Guadalupe Rodríguez Ferrante, Andrea Paula Goldin, Mariano Sigman, María Juliana Leone

**Affiliations:** 1 Laboratorio de Neurociencia, Universidad Torcuato Di Tella, CONICET, Av. Figueroa Alcorta, C1428BCW, CABA, C1428BIJ7350 Buenos Aires, Argentina; 2 Facultad de Lenguas y Educación, Universidad Nebrija, Madrid, Spain; 3 Laboratorio de Cronobiología, Departamento de Ciencia y Tecnología, Universidad Nacional de Quilmes, CONICET, Roque S. Peña 352, B1876BXD Bernal, Buenos Aires, Argentina

**Keywords:** Neuroscience, Psychology

## Abstract

The misalignment between late chronotypes and early school start times affect health, performance and psychological well-being of adolescents. Here we test whether, and how, the baseline chronotype (i.e. chronotype at the beginning of secondary school) and the school timing affect the magnitude and the direction of the developmental change in chronotype during adolescence. We evaluated a sample of Argentinian students (n = 259) who were randomly assigned to attend school in the morning (07:45 a.m.–12:05 p.m.), afternoon (12:40 p.m.–05:00 p.m.) or evening (05:20 p.m.–09:40 p.m.) school timings. Importantly, chronotype and sleep habits were assessed longitudinally in the same group of students along secondary school (at 13–14 y.o. and 17–18 y.o.). Our results show that: (1) although chronotypes partially align with class time, this effect is insufficient to fully account for the differences observed in sleep-related variables between school timings; (2) both school timing and baseline chronotype are independently associated with the direction and the magnitude of change in chronotype, with greater delays related to earlier baseline chronotypes and later school timings. The practical implications of these results are challenging and should be considered in the design of future educational timing policies to improve adolescents’ well-being.

## Introduction

Humans show close-to-24 h (or circadian) rhythms in their behavior and physiology. These fluctuations are endogenous and coordinated by a main circadian clock, located in the superchiasmatic nucleus^1^. Even though humans are active during daytime, the expression of the endogenous circadian timing under specific external conditions (i.e. chronotype) shows variability among individuals^2,3^. Chronotype has a genetic basis^4–6^, but it also depends on other factors, such as age^7–12^, light exposure^2,13,14^, and cultural and social cues^15–17^. Consistently, individuals’ chronotype ranges on a continuum between early and late types and can be assessed by evaluating behavioral^18–20^ or physiological rhythms^21–23^, as well as by using easy, reliable and standardized questionnaires. The widely used Munich Chronotype Questionnaire (MCTQ)^24^ assesses several sleep-related variables including a chronotype proxy based on sleep timing: the sleep-corrected midpoint of sleep on free days (MSFsc). This marker highly correlates with other measures, such as the Morning-Eveningness Questionnaire score^25–27^, the phase of actigraphy-evaluated sleep-rest activity rhythm^18,19,24^, core temperature and other endogenous physiological rhythms^21,22,28,29^.

Sleep timing approaches the individuals’ endogenous tendencies only on free days^18^, as opposed to weekdays, where sleep timing is usually influenced by working and education schedules^3^. Accordingly, modern societies are characterized by the prevalence of a misalignment between the biological timing and the social timing (i.e. determined by social cues). This chronic condition is known as social jetlag (SJL)^3,30^, which is calculated as the difference between the midpoint of sleep on free days and on weekdays^3,31^. It is worth noting that SJL refers to differences on sleep timing but not on sleep duration (SD). Importantly, both SJL and short SD have been associated with negative consequences for physical and mental health, such as obesity^31,32^, depression^33,34^, higher risk of substance abuse^35,36^, higher rates of suicide^36^, and impaired cognitive performance^37,38^.

Although adolescents’ chronotype is particularly late^7–12^, secondary school starts very early in the morning all around the world^39^. This contrast between biological timing and social obligations is proposed to be the main cause of short SD on weekdays (SDw) and high SJL during adolescence^34,35^. Consistently, delaying the school start time improves mood, wellbeing and academic performance^40–44^, and decreases diurnal somnolence and even the rate of vehicular accidents^40,41,45–47^. Altogether, these results suggest that a better alignment between adolescents’ internal clock and school schedule might improve both their health and performance.

As a social cue, school start time might potentially modulate adolescents’ chronotype. When comparing different morning school timings, results are not conclusive: a few studies found an association between later school start times and later chronotypes^48–50^, while others did not^51,52^. However, when more distant school timings were compared (e.g. morning vs. afternoon), later school timings were associated with later chronotypes^53–56^. Unfortunately, the lack of random assignment of students to different school timings might confound and/or mask the effect of school timing on chronotype (due to biased assignment based on academic performance, socio-economic status or chronotype preferences). Although it has been well established that chronotypes become progressively delayed until the end of adolescence^7–12^, whether and how this developmental effect is modulated by a social cue as school timing needs further investigation.

Recently, we partially addressed this point with a cross-sectional study comparing two well-defined age groups, younger and older adolescents randomly assigned to one of three different school timings at the beginning of secondary school: morning (07:45–12:05), afternoon (12:40–17:00) or evening (17:20–21:40)^57^. Taking advantage of this unusual random assignment, we studied the relationship between school timing, chronotype and age^55^. We found that, despite adolescents’ chronotype was partially aligned with their school timing, students continued to experience high SJL and short SDw. Importantly, school timing modulates these effects: morning-attending students presented the earliest chronotypes, but the highest SJL and the shortest SDw^57^. Our previous results also show an impact of age on chronotype, SJL and SDw, with larger differences between school timings for the oldest adolescents. The conclusions of this previous cross-sectional study regarding how the interaction between age and school timing affects chronotype are compelling, yet limited, because different students were evaluated at different time points during adolescence.

Here we present a follow-up study where the same group of students was evaluated at their 1st (13–14 y.o.) and their 5th (17–18 y.o.) year of secondary school. This longitudinal design allows us to assume that the differences observed between 1st and 5th year are due to age-related changes and not due to interindividual variability. First, we replicate, complement and strengthen the results obtained in our previous cross-sectional study (Hypotheses box H1). However, the novelty and main aim of the present study is to understand using a data-driven approach which factors modulate the developmental change in chronotype that occurs during adolescence (ΔChronotype = MSFsc_5th_—MSFsc_1st_, i.e. the variation in chronotype that occurs from 1st to 5th year of school. Note that a negative ΔChronotype implies a chronotype advance, i.e. a later chronotype in 1st than in 5th year. In contrast, a positive value implies a chronotype delay). Importantly, the assessment of this aim is only possible with data corresponding to different time points for the same adolescents.

**Figure 1:**
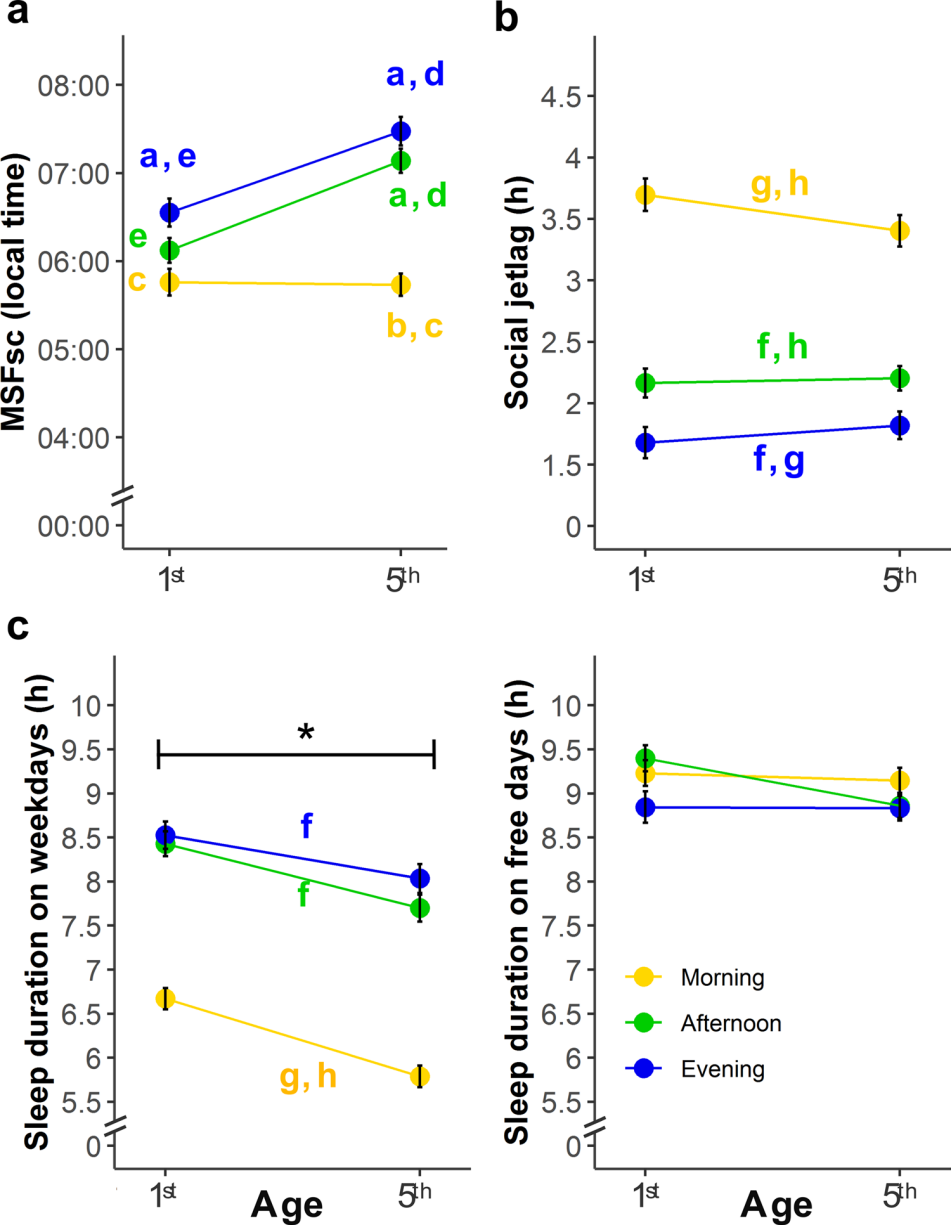
Longitudinal changes in Chronotype, Social jetlag and Sleep duration during adolescence. (**a**) Mean changes on Chronotype depend on school timing and age. Evening-attending students exhibit later MSFsc than their morning-attending peers: 47 min in 1st year (06:33 vs. 05:46) and it doubles to 104 min in 5th year (07:28 vs. 05:44). Afternoon-attending students show a similar pattern in 5th year: 82 min later MSFsc than their morning-attending peers (07:08 vs. 05:46). Post-hoc pairwise comparisons, *P* < 0.006 (Bonferroni-corrected *P* < 0.05). (**b**) SJL depends on school timing. SJL levels are lower for evening-attending students than for their afternoon-attending peers, both in 1st year (1.68 h vs. 2.16 h) and in 5th year (1.80 vs. 2.20). The same happens when compared to morning-attending students, who present the highest SJL levels (3.70 h and 3.40 h in 1st and 5th year, respectively). No significant differences were found between 1st and 5th year at any school timing. Post-hoc comparisons, *P* < 0.017 (*P* < 0.05, Bonferroni corrected). (**c**) School timing and age affect sleep duration on weekdays (SDw) but not on free days (SDf). On weekdays, adolescents sleep less in their 5th year regardless of their school timing, and in the morning school timing regardless of their age. Students sleep more on free days than on weekdays independently of their age and school timing. No differences were found between school timings and age on free days. The interaction between school timing and age was not significant. The asterisk (*) indicates significant difference in sleep duration between 1st and 5th year across school timings, which was found on weekdays but not on free days. Post-hoc comparisons, *P* < 0.0038 (*P* < 0.05, Bonferroni corrected). Data are Mean ± s.e.m. N = 259. Lowercase letters indicate significant differences between groups: (**a**) compared with morning of the same school year; (**b**) compared with afternoon of the same school year; (**c**) compared with evening of the same school year; (**d**) compared with 1st year of the same school timing; (**e**) compared with 5th year of the same school timing; (**f**) compared with morning, across age groups; (**g**) compared with afternoon, across age groups; (**h**) compared with evening, across age groups.

As mentioned before, it has been reported that chronotype is modulated by social cues^15–17^ and age^7,8^ among other factors^2,54,57,58^. However, some recent studies found low-to-moderate stability throughout adolescence in chronotype at individual level^9–11^. This result means that even though chronotype delays on average, the developmental change (ΔChronotype) is not necessarily the same for all students. Factors explaining this variability in ΔChronotype are unknown. Here, we propose that this change is not only modulated by school timing, but also it is correlated with baseline chronotype (i.e. the chronotype of each student in 1st year). The relationship between these two factors leads to four possible and alternative scenarios, where the magnitude and/or the direction of ΔChronotype is associated with school timing and/or each adolescent’s baseline chronotype (Hypotheses box H2, Box Fig. 1, Supp. Fig. 1).

Although the four scenarios are possible, we know that: 1- Argentinian adolescents exhibit later chronotypes than adolescents from other countries^7,8,53,64–66^, and their baseline chronotypes are particularly late; and 2- school timing, as a social cue, has been shown to modulate chronotype^54,57^. Consistently, we think that the independent association of both school timing and baseline chronotype will better explain the age-related changes in chronotype (i.e. Hypotheses box H2, scenario c). Importantly, as the interplay of late chronotypes and early school start times is expected to be the cause^39,64^ of unwanted sleep-associated conditions (e.g., short SD and high SJL^39,64–69^), we hypothesize that changes in social jetlag and sleep duration will be associated with ΔChronotype and influenced by school timing (Hypotheses box H3). Particularly, we predict that a higher ΔChronotype in morning-attending students will be associated with even higher SJL and lower SDw in 5th year, compared with other school timings. In summary, we expect that both school timing and baseline chronotype will be associated with the magnitude and direction of ΔChronotype that, in turn, will be associated with changes in levels of SJL and SDw during adolescence.

Hypotheses box **H1**

*Longitudinal changes in chronotype, sleep duration and social jetlag are similar to the changes we previously observed in our cross-sectional study*. Briefly, we predict later chronotypes when adolescents are older and attend to later school timings (i.e. afternoon and evening). Additionally, we expect higher social jetlag and shorter sleep duration when adolescents are older and attend to earlier school timings (e.g. morning).

**H2**

*Both school timing and baseline chronotype are independently associated with the developmental change in chronotype.* Previous cross-sectional studies showed that chronotype is progressively delayed during adolescence until it reaches a peak at the end of this developmental stage^7–12^. Under comparable environmental cues (e.g. light–dark cycle), this chronotype delay should have limits: either because of the magnitude of the change and at what age this ‘delaying process’ starts and ends during adolescence, or because the limits imposed by the intrinsic mechanism of the circadian clock^17,59–63^. Importantly, individuals will not be entrained to the environment outside these theoretical upper (and lower) limits of chronotype (Supp. Fig. 1). The exact value of the upper limit is unknown and might depend on different environmental and social factors (e.g. light exposure, geographical longitude or latitude, culture, etc.). Beyond that, depending on the limit value, the magnitude and/or the direction of the developmental change in adolescents’ chronotype will (or will not) be affected. For example, an extremely late upper limit will have no effect on adolescents’ chronotype (because adolescents will not reach that upper limit, even at the end of secondary school).

Four alternative scenarios appear when considering the previously mentioned upper limits and including school timing and baseline chronotype as independent variables in a correlation model explaining the magnitude and/or the direction of the developmental changes of chronotype during adolescence.

- The school timing affects the magnitude (not the direction) of ΔChronotype independently of each student's baseline chronotype (Box Fig. 1a). Earlier school timings will exert more pressure on the age-associated delay in chronotype. Thus, the magnitude of ΔChronotype will be smaller in students attending the morning school timing than in students in the other school timings. Note that in this scenario the age-associated expected change does not exceed the upper limit of chronotype (Supp. Fig. 1a).
- Only the baseline chronotype, and not school timing, is related to the magnitude (but not the direction) of ΔChronotype (Box Fig. 1b). Students with later baseline chronotypes will reach the upper limit of the possible chronotypes range before their peers with earlier baseline chronotypes. Consequently, the magnitude of ΔChronotype will be smaller in students with later baseline chronotypes, irrespective of school timings. Moreover, as students became older, chronotypes would be delayed for all school timings. Note that in this scenario the theoretical upper limit has to be lower than in scenario 1, affecting students’ ΔChronotype (Supp. Fig. 1b).

**Box Figure 1:**
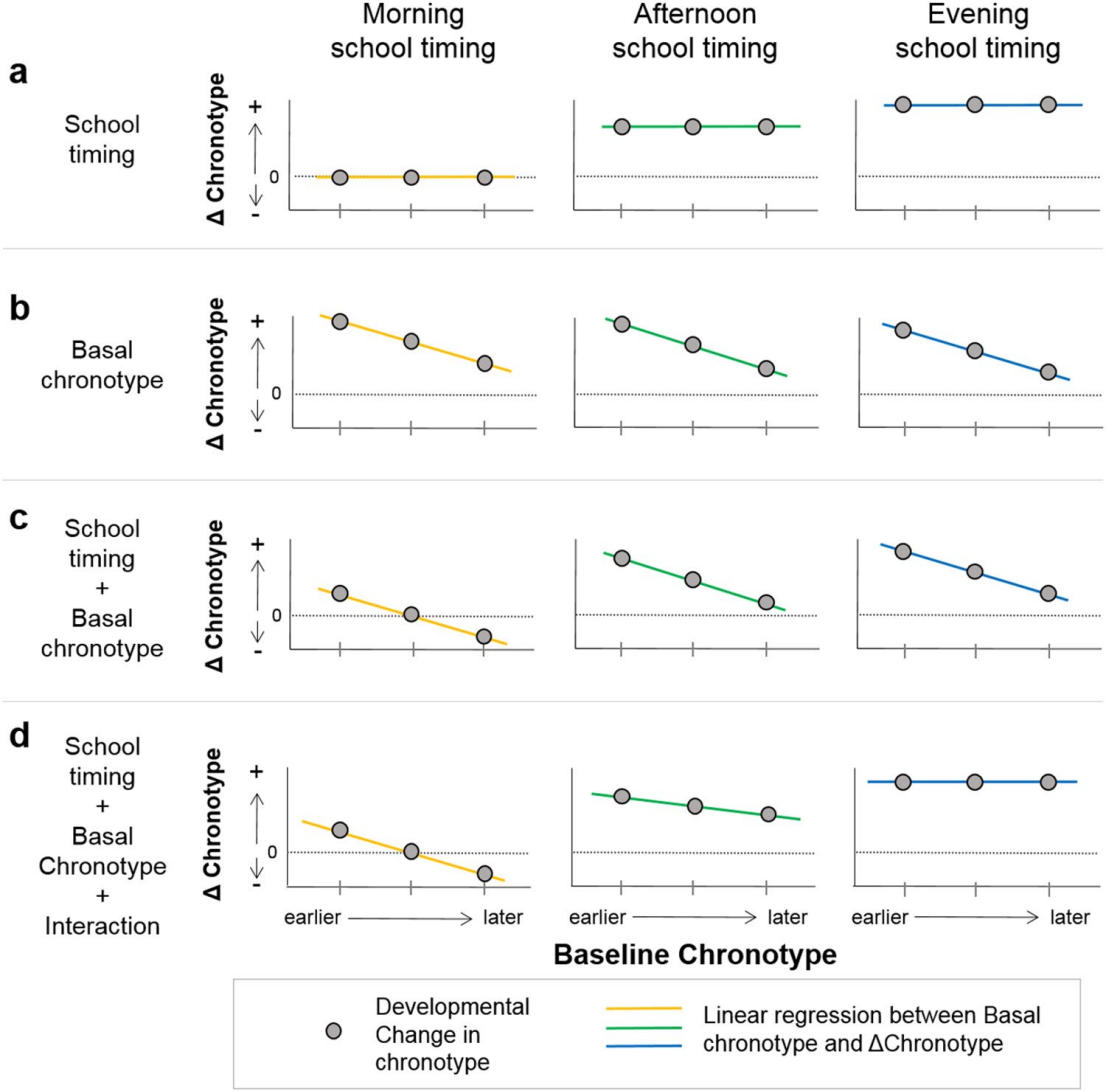
Four theoretical scenarios of the association of school timing and/or baseline chronotype (in 1st year of high school) with ΔChronotype (MSFsc 5th year-MSFsc 1styear). (**a**) Only school timing is associated. (**b**) Only the baseline chronotype is associated. (**c**) Baseline chronotype and school timing are additively associated. (**d**) Baseline chronotype and school timing interact. The graphs on each scenario represent the expected developmental change in chronotype (i.e. ΔChronotype) as a function of the baseline chronotype (i.e. 1st year MSFsc) for each school timing. A zero value on the vertical axis indicates no change in chronotype from 1st to 5th year. Positive or negative values indicate that chronotype is delayed or advanced, respectively, in 5th compared to 1st year. Each colored line represents the linear relation between ΔChronotype and baseline chronotype for each school timing. Grey arrows represent ΔChronotype for three representative baseline chronotypes (early, intermediate and late), the base of the arrows represent students’ chronotype in their 1st year (i.e. the baseline chronotype) and the arrowheads represent students’ chronotype in their 5th year.
- School timing and baseline chronotype are associated with the magnitude and direction of ΔChronotype. Here, both phenomena act together but independently (Box Fig. 1c). On the one hand, later baseline chronotypes will experience a smaller ΔChronotype due to reaching the upper limit of possible chronotypes. On the other hand, school timing will also affect ΔChronotype: earlier school timings will exert higher pressure and, consistently, students attending school earlier will experience smaller ΔChronotype. Note that the school timing effect would take place considering the existence of the upper limit and, in the most extreme cases, it would lead to negative ΔChronotype (i.e. morning attending students with late baseline chronotypes will not delay, or will even advance, their chronotype, experiencing an earlier chronotype in their 5th year compared to their 1st) (Supp. Fig. 1c).
- Not only school timing and baseline chronotype but also their interaction are associated with the magnitude and the direction of ΔChronotype (Box Fig. 1d). In this scenario, the age-associated expected change in chronotype is within the range of possible chronotypes. Each school timing differentially affects ΔChronotype depending on the baseline chronotype. In particular, while the magnitude of the pressure exerted by morning and afternoon school timing is larger for later chronotypes, the evening school timing is late enough to not exert any pressure, regardless of baseline chronotype (Supp. Fig. 1d).

**H3**

*Age-related changes in both social jetlag and sleep duration are associated with ΔChronotype and modulated by school timing.* We predict that larger chronotype delays would be associated to higher levels of social jetlag and shorter sleep duration on weekdays, with stronger associations in the morning school timing.

## Results

### Mean chronotype and SDw depend on school timing and age, while SJL depends only on school timing (H1)

To evaluate how school timing and age longitudinally affect chronotype during adolescence (data distribution in Supp. Fig. 2), we ran a linear mixed-effect model with chronotype (i.e. MSFsc) as the dependent variable, including school timing (morning, afternoon or evening), age (1st or 5th year) and their interaction as fixed effects, and students’ id as a random effect (Supp. Table 1, Supp. Table 2). As in our previous cross-sectional results^57^, we found a main effect of school timing (F_2,256_ = 29.697, *P* < 0.0001, partial *η*^2^ = 0.188, 90% confidence interval (CI) = 0.119–0.256). Morning-attending students presented earlier chronotypes than both afternoon- and evening-attending students (Fig. 1a, Supp. Table 3), suggesting that school timing affects students’ biological time, improving its alignment to the school timing where students were randomly assigned. We also found a significant main effect of age (F_1,256_ = 41.921, *P* < 0.0001, partial *η*^2^ = 0.141, 90% CI = 0.081–0.207), with earlier chronotypes in 1st year. Importantly, a significant interaction between school timing and age (F_2,256_ = 12.062, *P* < 0.0001, partial *η*^2^ = 0.086, 90% CI = 0.036–0.142) reveals that chronotype’ changes throughout adolescence are modulated by school timing. At 1st year, adolescents’ chronotype only slightly differed between school timings, but this difference gets larger by their 5th year (Fig. 1a). Consistently with our previous cross-sectional study, school timing modulates how adolescents’ chronotype changes with age.

To evaluate whether the observed modulation was sufficient to fully, or only partially, align students’ chronotype with their school schedules, we assessed the effects of age and school timing on both social jetlag (SJL) and sleep duration (SD) levels.

First, we ran a mixed effect model for SJL including school timing and age (data distribution in Supp. Fig. 3) as fixed factors and students’ id as a random effect (Supp. Table 4, Supp. Table 5). School timing significantly affects SJL (F_2,256_ = 97.691, *P* < 0.0001, partial *η*^2^ = 0.433, 90% CI = 0.360–0.496), but we did not find significant effects neither for age (F_2,256_ = 0.194, *P* = 0.660, partial *η*^2^ = 0.001, 90% CI = 0.000–0.016) nor for the interaction between age and school timing (F_2,256_ = 2.288, *P* = 0.104, partial *η*^2^ = 0.018, 90% CI = 0.000–0.048). Particularly, morning-attending adolescents present significantly higher SJL levels (close to 3.5 h) than their peers attending later school schedules (Fig. 1b, Supp. Table 6). In addition, afternoon-attending students present higher SJL levels than their evening-attending peers (2.18 h vs. 1.74 h), suggesting that afternoon school schedules also exert pressure on adolescents’ sleep timing. Results show similar main trends when correcting the formula of social jetlag for sleep debt^70^.

Second, we ran a mixed effect model for Sleep Duration (SD) including school timing, age and type of day (weekday or free day) (data distribution in Supp. Fig. 4 and sleep timings in Supp. Table 7) as fixed effects and students’ id as a random effect (Supp. Table 8, Supp. Table 9). We found significant main effects for school timing (F_2,256_ = 32.059, *P* < 0.0001, partial *η*^2^ = 0.200, 90% CI = 0.130–0.313), age (F_2,768_ = 35.032, *P* < 0.0001, partial *η*^2^ = 0.044, 90% CI = 0.023–0.070) and type of day (F_2,768_ = 392.264, *P* < 0.0001, partial *η*^2^ = 0.338, 90% CI = 0.276–0.379). This last effect indicates that students sleep less on weekdays, as expected. Additionally the interactions between type of day and age (F_2,768_ = 10.117, *P* = 0.002, partial *η*^2^ = 0.013, 90% CI = 0.003–0.029) or school timing (F_2,768_ = 90.161, *P* < 0.0001, partial *η*^2^ = 0.190, 90% CI = 0.150–0.230) were also significant. Conversely, the interaction between age and school timing (F_2,768_ = 1.998, *P* = 0.136, partial *η*^2^ = 0.005, 90% CI = 0.000–0.015) and the triple interaction between age, school timing and type of day (F_2,768_ = 1.368, *P* = 0.255, partial *η*^2^ = 0.004, 90% CI = 0.000–0.012) were not significant. On weekdays, students sleep less when they are older and morning-attending students sleep less than adolescents with later school schedules (Fig. 1c). Instead, students do not differ in their sleep duration, despite their age or school schedule, on free days (Fig. 1c, Supp. Table 10).

Thus, morning-attending students present very short SDw (i.e. high levels of sleep loss) in their 1st year of school and this situation aggravates as adolescence progresses. The difference in SDw between school timings was not compensated by napping (Supp. Fig. 5, Supp. Table 11, Supp. Table 12, Supp. Table 13): even considering naps, morning-attending students do not reach the recommended 8 h of sleep^71–73^. Altogether, the results presented here support that school timing modulates only partially adolescents’ chronotype with both SJL and SDw levels depending on the school timing to which students were randomly assigned at the beginning of secondary school.

### The developmental change in chronotype during adolescence is associated with both school timing and baseline chronotype (H2)

In the previous section, we showed that mean changes in chronotype depend on age and school timing. However, the association between 1st and 5th year’s chronotypes is low-to-moderate in all school timings (morning: t = 3.462, *P* = 0.001 r = 0.344, 95% CI = 0.149–0.514; afternoon: t = 1.769, *P* = 0.080 r = 0.182, 95% CI = − 0.022–0.372; evening: t = 4.439, *P* < 0.0001 r = 0.461, 95% CI = 0.261–0.623) (see also Supp. Fig. 6). Here, we propose that baseline chronotype (i.e. 1st year chronotype) might be related to this lack of stability. Consistently, we contrasted the four scenarios previously described (Hypotheses box H2, Box Fig. 1, Supp. Fig. 1) to evaluate whether baseline chronotype and school timing are associated with the developmental changes in chronotype (i.e. age-related changes in chronotype or ΔChronotype).

**Figure 2:**
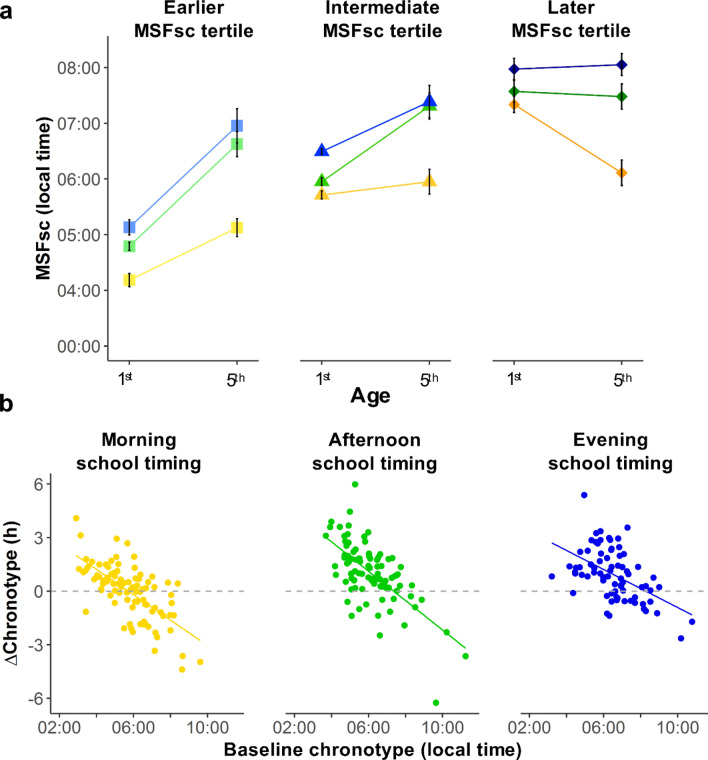
Developmental changes in chronotype (ΔChronotype) during adolescence is associated with both school timings and students’ baseline chronotype. (**a**) Baseline chronotype tertile affects the change in Chronotype from 1st to 5th year. Those students on the earliest tertile delayed their chronotype by their 5th year, independently on school timing (from 04:11 to 05:08, from 04:47 to 06:38, from 05:08 to 06:58, for morning-, afternoon- and evening- attending students respectively). Students on the latest tertile of MSFsc, on the other hand, did not delay or even advanced their chronotypes (from 07:20 to 06:07, from 07:34 to 07:29, from 07:59 to 08:03, for morning-, afternoon- and evening- attending students respectively). (**b**) ΔChronotype correlates with baseline chronotype and school timing. Students with a late baseline MSFsc experienced a lower chronotype change from 1st to 5th year. Particularly, the slope of the relationship between ΔChronotype and baseline chronotype indicates that, when the baseline chronotype is 1-h later, the age-related changes on chronotype are lower: 43 min (95% CI = − 53 to − 32 min), 49 min (95% CI = − 61 to − 38 min) and 32 min (95% CI = − 44 to − 20 min) for morning-, afternoon- and evening-attending students, respectively. N = 259. ΔChronotype = MSFsc 5th year—MSFsc 1st year. Baseline chronotype = MSFsc in 1st year. Color indicates school timing: yellow, green and blue for morning, afternoon and evening, respectively.

Baseline chronotype tertiles suggest that the developmental change in chronotype is associated with baseline chronotype, with adolescents in the earliest tertile delaying their chronotypes the most during secondary school (Fig. 2a, Supp. results). We ran a linear regression model with ΔChronotype (MSFsc 5th year—MSFsc 1st year) as the dependent variable, and both school timing and baseline chronotype, as predictors (Supp. Table 14, Supp. Table 15). ΔChronotype was associated with both school timing (F_2,253_ = 19.678, *P* < 0.0001, partial *η*^2^ = 0.135, 90% CI = 0.073–0.198) and baseline chronotype (F_1,253_ = 160.343, *P* < 0.0001, partial *η*^2^ = 0.388, 90% CI = 0.314–0.455). ΔChronotype was smaller for later than for earlier baseline chronotypes, meaning that chronotype becomes less delayed with age for later baseline chronotypes (Fig. 2b). For all school timings, the slopes of the relationship between the ΔChronotype and the baseline chronotype were different to zero (Morning: b = − 0.711, 95% CI = − 0.888 to − 0.534, t = − 7.909, *P* < 0.0001; Afternoon: b = − 0.823, 95% CI = − 1.009 to − 0.637, t = − 8.717, *P* < 0.0001; Evening: b = − 0.533, 95% CI = − 0.738 to − 0.327, t = − 5.109, *P* < 0.0001). However, the interaction between baseline chronotype and school timing was non-significant (F_2,253_ = 2.150, *P* = 0.119, partial *η*^2^ = 0.017, 90% CI = 0.000–0.037), showing that slopes did not differ between school timings. Thus, the magnitude of ΔChronotype is similarly related to baseline chronotype for the three school timings. For example, a 1st year afternoon-attending student with a baseline chronotype equal to the mean for the afternoon school timing (MSFsc = 06:07) would delay their chronotype by 61 min by the time s/he gets to 5th year. However, a same-class peer with a baseline chronotype of 07:07 (1 h later) would only delay it 12 min (i.e. the difference between these students**’** ΔChronotype is 49 min, which is the slope of the model for the afternoon).

Our results are consistent with scenario c (Hypotheses Box H2): both school timing and baseline chronotype are additively associated with ΔChronotype during adolescence, with no interaction between them. Even though morning-attending students experienced, on average, a lower delay in their chronotype from 1st to 5th year (compared with their afternoon- and evening-attending peers), overall, students with earlier baseline chronotypes exhibited larger delays and those with later chronotypes showed smaller delays or advances, regardless of their school timing.

### Age-related changes in SJL and SDw are associated with ΔChronotype and school timing (H3)

**Figure 3:**
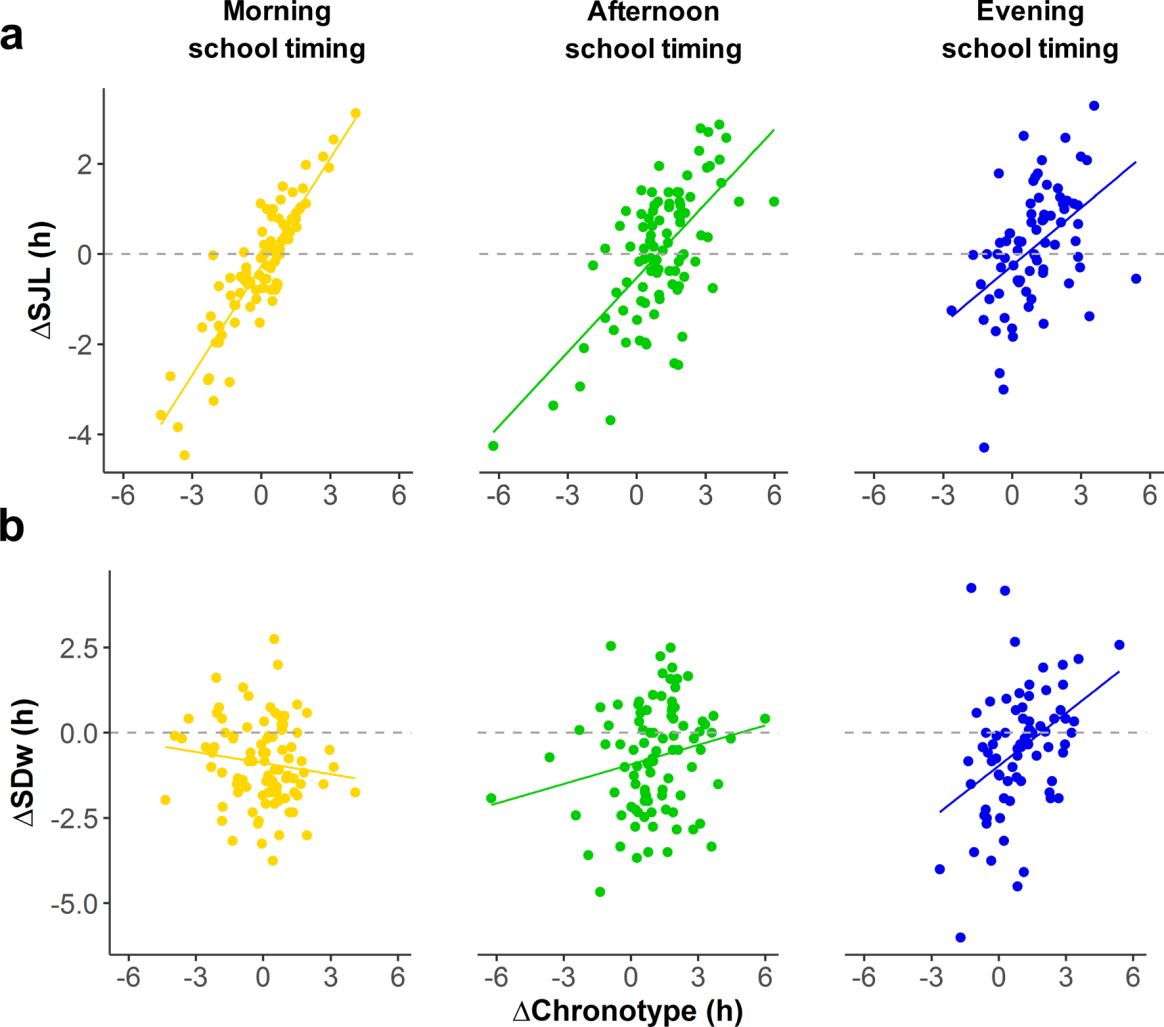
Age-related changes in Social jetlag and Sleep duration on weekdays are associated with developmental changes in chronotype. (**a**) ΔSJL (SJL 5th year—SJL 1st year) highly correlates with ΔChronotype (MSFsc 5th year—MSFsc 1st year). Higher increases in SJL levels are associated with larger delays on chronotype from 1st to 5th year (i.e. slopes): morning-, afternoon- and evening-attending students experience an increase of 48 min (95% CI = 40–56 min), 33 min (95% CI = 26–40 min) and 26 min (95% CI = 16–35 min), respectively, in SJL for each hour that their chronotype is delayed. (**b**) ΔSDw (SDw 5th year—SDw 1st year) association with ΔChronotype depend on school timing. For morning-attending students, the slope indicates that a 1 h-delay in chronotype from 1st to 5th year leads to a 7 min decrease (95% CI = − 19–6 min) in SDw, but it does not significantly differ from zero. This tendency is inverted and significant for afternoon- and evening-attending students, who increment their sleep in 11 min (95% CI = 1–22 min) and 31 min (95% CI = 17–45 min), respectively, for each hour their chronotype is delayed. N = 259.

Later chronotypes are associated with higher levels of social jetlag (SJL) and a lower sleep duration on weekdays (SDw), especially when attending school in the morning^39,65–69^. Here we explored whether the individual changes in SJL or SDw during adolescence depend on ΔChronotype and/or school timing. We ran a linear regression model with the age-related changes on SJL (i.e. ΔSJL = SJL 5th year—SJL 1st year) (Supp. Fig. 7) as the dependent variable and ΔChronotype and school timing as predictors (Supp. Table 16, Supp. Table 17). We found significant main effects of both school timing (F_2,253_ = 4.493, *P* = 0.012, partial *η*^2^ = 0.034, 90% CI = 0.004–0.075) and ΔChronotype (F_2,253_ = 235.795, *P* < 0.0001, partial *η*^2^ = 0.482, 90% CI = 0.413–0.543) (similar results were found when using SJL sleep corrected formula^70^). In brief, the more delayed the chronotype becomes from 1st to 5th year, the bigger the change in SJL. For example, if a hypothetical afternoon-attending student exhibits a ΔChronotype equal to the mean change for their school timing (ΔChronotype = 61 min, e.g. from 05:00 to 06:01), their SJL will increase by just 2 min. However, another student, with a 1 h-larger ΔChronotype (e.g. from 05:00 to 07:01, i.e. 121 min), would increase their SJL on 35 min along secondary school. Importantly, the interaction between ΔChronotype and school timing was significant (F_2,253_ = 7.021, *P* = 0.001, partial *η*^2^ = 0.053, 90% CI = 0.014–0.100). The association between ΔSJL and ΔChronotype was progressively weaker the later the school timing, even though the comparison between afternoon and evening school timings was not significant (Fig. 3a, Supp. Table 18). Morning-attending students exhibit larger changes in SJL for a given ΔChronotype, compared with their afternoon- and evening-attending peers (slope comparisons: morning vs. afternoon: t = 2.767, *P* = 0.017; morning vs. evening: t = 3.552, *P* = 0.001).

Age-related changes in SDw also showed interindividual differences (Supp. Fig. 8), even though changes on mean SDw were similar when comparing school timings (Fig. 1c). We ran a linear regression model with the age-related changes in SDw (ΔSDw = SDw 5th year—SDw 1st year) as the dependent variable, and ΔChronotype and school timing as predictors (Supp. Table 19 Supp. Table 20). As expected, the main effect of school timing was non-significant (F_2,253_ = 1.433, *P* = 0.241, partial *η*^2^ = 0.011, 90% CI = 0.000–0.037), indicating that SDw change similarly in different school timings (Fig. 1c). We found a significant main effect of ΔChronotype (F_1,253_ = 8.196, *P* = 0.0046, partial *η*^2^ = 0.031, 90% CI = 0.006–0.075) and, importantly, a significant interaction between ΔChronotype and school timing (F_2,253_ = 7.852, *P* < 0.001, partial *η*^2^ = 0.058, 90% CI = 0.017–0.108), indicating that school timing modulates the effect of ΔChronotype on age-related changes in SDw. In particular, afternoon- and evening-attending students with larger delays in their chronotype throughout adolescence exhibit less shortening, or even a lengthening, of their SDw (afternoon: b = 0.191, 95% CI = 0.013–0.369, t = 2.108, *P* = 0.036; evening: b = 0.515, 95% CI = 0.278–0.756, t = 4.281, *P* < 0.0001) (Fig. 3b). To illustrate, an average afternoon-attending student (ΔChronotype = 61 min) would decrease their SDw by 44 min, while a peer with a 1 h-larger ΔChronotype (i.e. 121 min) would decrease their SDw by 32 min. Note that the corresponding slope is the difference between 44 and 32 min, which is 12 min. On the other hand, morning-attending students with the greatest delays in their chronotypes by their 5th year, showed a tendency to shorten their SDw the most, although the slope was not different from zero (b = − 0.109, 95% CI = − 0.310–0.093, t = − 1.062, *P* = 0.289). Despite the fact that both the slopes for afternoon- and evening-attending students did differ from zero, only evening and morning slopes significantly differ between them (morning vs. evening: t = − 3.950, *P* < 0.001) (Supp. Table 21). Even though one would expect that age-related chronotype delays in morning-attending adolescents would be strongly associated with a comparable increase in SJL and decrease in SDw^3,54,74–76^, our results show that SJL increases accordingly with the chronotype delay while SDw did not decrease as much as expected.

## Discussion

Here we achieved three related and complementary aims. First, we reproduced and strengthened our previous cross-sectional results^57^ on how school timing and age affect chronotype and sleep. Second, we showed that the magnitude and the direction of the age-associated change in chronotype is associated with both school timing and baseline chronotype (i.e. chronotype of adolescents in their 1st year of secondary school) consistent with scenario c (Hypotheses box H2, Box Fig. 1c). We proposed a mechanism that is consistent with our results (i.e. the existence of an upper limit), but our data is not sufficient to test which is this limit or if other mechanism is involved. Third, we found that the developmental changes in chronotype are associated with social jetlag and sleep duration age-related changes.

Consistently with our previous data^57^, we found that students’ chronotypes were partially aligned with their school timing. Chronotype depends on both school timing and age, as well as on their interaction: the midpoint of sleep on free days (MSFsc) is later in older adolescents and later school timings, with larger differences between school timings for older adolescents. Social jetlag (SJL) is higher and sleep duration on weekdays (SDw) is shorter when school timing is earlier, especially for older students attending school in the morning. Most results were consistent between both studies, reinforcing our conclusion that school timing, as a social cue, partially modulates adolescents' internal timing.

Importantly, our longitudinal design allowed us to further analyze the low-to-moderate stability of chronotype during adolescence reported in previous longitudinal studies^9–11^. We found that ΔChronotype correlates with baseline chronotype. A previous study reported that chronotype development was associated with the interaction between age and circadian preferences^9^ finding similar results. However, baseline chronotype as a factor related to the low stability of chronotype along development has not been previously reported and, thus, our approach and results contribute to understand how chronotype changes during adolescence.

Previous works have studied how chronotype is affected either by school timing^7,8,62^ or by age^7–12,60^ during adolescence, but here we analyzed these two factors together and longitudinally. Our results are consistent with our prediction that baseline chronotype and school timing are independent and additive associated with adolescents’ chronotype (Supplementary Fig. 1c, Scenario 3): 1- later school timings are related to later chronotypes, with a stronger association in older adolescents, and 2- earlier baseline chronotypes experience a bigger ΔChronotype. According to our model, while a morning-attending student with a baseline chronotype equal to 05:46 (i.e. the mean baseline chronotype for this school timing) practically does not change their chronotype during secondary school, 1-h later baseline chronotypes (i.e. 06:46) would advance their chronotypes 45 min by 5th year. Thus, morning-attending adolescents who have late baseline chronotypes at the beginning of secondary school might experience smaller delays or even advance their chronotype with age. As summarized in Scenario 3, getting older is not necessarily associated with later chronotypes.

Here we propose a mechanism that includes the existence of limits to ΔChronotype during adolescence. Particularly, an upper limit associated with either the developmental stage achieved at the end of secondary school^59,60^ or with the entrainment mechanism of the circadian clock^62^ might explain the association of both school timing and baseline chronotype with ΔChronotype. Previous works showed that chronotype variability among adolescents depends on different factors, including genetics, culture, light exposure, schedules and age^2,4–8,12–17,57^. On the one hand, advanced pubertal stages have been associated with later chronotypes^59,80^ students with later baseline chronotypes might be the ones presenting the most advanced pubertal stages at the beginning of secondary school. If this case, they would reach the upper limit before their peers and, consistently, they would have a smaller delay in chronotype between 1st and 5th year than they peers who started secondary school at lower pubertal stages. On the other hand, the upper limit might be associated with the entrainment mechanism. To be entrained to the external 24 h light–dark cycle, humans have to be exposed to light at specific times of the day^2,17,62^. Consistently, the interindividual variability exists but has limits and the range of chronotypes does not cover the 24 h (i.e. some theoretically possible chronotypes might not be compatible with entrained rhythms^62^). Although humans can invert their wake-sleep cycle to be active at night and sleep during the day, as individuals who work night shifts, these subjects do not exhibit stable entrained rhythms^61,63,81^. As chronotype is delayed throughout adolescence^7,8,10,11^, students with later baseline chronotypes would reach the upper limit of stable entrainment^82^ before their earlier peers. Furthermore, in our setting, this scenario is especially plausible because Argentinian adolescents exhibit particularly late baseline chronotypes^7,8,53,57,82,83^. Based on our data, we cannot disambiguate whether the upper limit of chronotype exists and depends on the developmental stage and/or on the entrainment mechanisms of the circadian clocks. A possible concern associated with the correlation between the changes observed in MSFsc and its baseline value is that it might be partially/fully explained by the regression towards the mean^84^. This effect occurs because there exists random errors when a variable is assessed within-subjects. Thus, if the initial measurement were an extreme value it is highly probable that the next time we assess the same variable in the same subject the value would be closer to its true mean. This could lead to an artificial association between baseline values and the change observed in the variable. Consistently, the magnitude of the effect depends on the magnitude of within-subject variability and on the precision of the measurement instrument used. Although we acknowledge that this artifact might be influencing our results (especially considering that there is scarce literature validating MCTQ in adolescents), the effect of the regression towards the mean, if present, would only partially affect our results and it does not detract them from its validity. First, MCTQ is a reliable instrument to assess adolescents’ chronotype: similar results (magnitude and direction) associated with age and school timing were obtained using MCTQ^8,54,57,85,86^ and other instruments (e.g. DLMO, actigraphy, MEQ, etc.)^7,9,10,41,86–88^. Second, MCTQ test–retest reliability is good when assessing adolescents and young adults^89^ and ΔChronotype does not change when adolescents are evaluated and reevaluated with one year difference; furthermore, no differences were found between early and late types^90^. That is, variability within-subjects seems to be low. Moreover, similarly to the results presented here, when the period between test and retest during adolescence is longer, the midpoint of sleep derived from actigraphy showed higher within-subject variability^9,10^. Altogether, these findings support the idea that our results are due to a development effect that is built during adolescence and not due to regression towards the mean derived from MCTQ lack of reliability. Finally, we reported a difference of almost 2 h in ΔChronotype when comparing earlier and later chronotypes on different school timings (Fig. 2a). This magnitude is biologically significant and it is highly improbable that it would be observed only because of regression towards the mean, especially when well established effects acting on chronotype during adolescence, such as age and school timing effects, rarely reach this magnitude^8–10,41,54,57,87^. Future work is needed to fully understand the causes of the association we found between baseline chronotype and ΔChronotype during adolescence.

Importantly, our results show not only that ΔChronotype correlates with baseline chronotype and School timing but also that ΔChronotype is associated with age-related changes in SJL and SDw. As expected, large delays in chronotype were associated with an increase in SJL and this association progressively weakens from morning to evening school timings (Supp. Discussion). In contrast, a delay in chronotype was associated with an increase in SDw in both afternoon and evening school timings, with a steeper association for the latter, and we found no association in the morning (Supp. Discussion). Thus, a better alignment between adolescents’ internal timing and school timing seems to be beneficial in terms of sleep duration for afternoon- and evening-attending adolescents but not for their morning-attending peers. The latter was surprising because, although most adolescents shorten their SDw from 1st to 5th year, the difference on SDw does not depend on their ΔChronotype. A possible explanation for this result is that morning-attending students were already sleep-deprived in their 1st year and, consequently, their SDw might not be further shortened because of homeostatic reasons. Thus, the effect of a delay in Chronotype in morning-attending students is mostly absorbed by an increased in SJL levels and not by a shortening on SDw.

This study has several limitations. First, chronotype and sleep-related variables were self-reported through standardized questionnaires. Consistently, we cannot rule out a bias in students' answers, but they are highly improbable because students were blind to our experimental hypotheses. However, objective assessment of sleep and chronotype, such as actigraphy, could be more suitable. Second, our analyses are based on regressions, which do not allow us to establish causality but only association among variables. Third, we did not have access to other predictors that might modulate chronotype and its developmental change, such as pubertal stage, socioeconomic status, the usage of medications, the presence of illnesses, etc. Fourth, in this longitudinal study we only have data from two time points, one at the first and another at the last year of secondary school. The inclusion of additional time points (e.g. in the middle of secondary school) would be preferable but it was impossible due to operative reasons. Finally, the lack of assessment of chronotype and sleep habits before the beginning of secondary school does not allow us to unequivocally affirm that the initial point was completely balanced between school timings, even considering the random assignation.

This research also has some important strengths. First, the longitudinal design allows us to study the developmental changes during adolescence. Second, the sample size of our study is one of the highest among similar studies^53,54,56,91,92^. Third, as in our previous study, we worked with three different school schedules, including an evening school timing (17:20 – 21:40). Finally, the random assignation of students to a particular school schedule at the beginning of their secondary school, allowed us to assume no differences in chronotype and sleep habits between school timings before starting secondary school.

Our results have several practical implications. First, we found that an early morning but also the afternoon school timing is associated with unhealthy sleep habits in adolescents. Consistently, a practical implication when thinking about better school start times would be that later morning school starting times might help but may not be enough for adolescents to have healthy sleep. This is especially relevant in populations exhibiting particularly late chronotypes, such as the adolescents from Argentina, Uruguay and Spain^54,93^. In these situations, an evening school timing might be at least considered by the educational community and/or policy makers. Many families and even the educational community believed that the morning school timing is 'the most favorable school timing', but our work and several others support the idea that it is not the case: afternoon (or evening) school timing might be a more equitable and even preferable environment where early chronotypes do not present an advantage over late ones^53,55,57^. Second, we show that school timing modulates chronotype and sleep habits during adolescence and, then, the undesirable conditions or behaviors associated with eveningness reported in the literature (e.g. depressive feelings or substance use) might be associated with the lack of alignment between chronotype and school timing (previous studies only include students that attend school in the morning^10,11,94–99^). Thus, future studies should include the effect of school timing. Third, the low-to-moderate stability observed in chronotype during adolescence suggests that chronotype is a malleable target for interventions^10,11,94–99^: knowing that earlier baseline chronotypes would exhibit larger delays, provides us new insights to help design interventions addressing adolescents sleep health and behavior.

Finally, the association between baseline chronotype and the magnitude and direction of ΔChronotype reported here go against the most parsimonious and intuitive notion that all adolescents would similarly delay their chronotype. Chronotype does not necessarily delay during adolescence and it is associated with baseline chronotype and school timing. These results might modify the previously suggested policies to improve the alignment between school timing and adolescents' internal timing. For example, the assignment of school timing based only on baseline chronotype would not be as beneficial for adolescents' sleep health and academic performance^53–55,57^ as expected. Of course, more evidence is needed to shed light on this matter and to understand their practical implications, but this novel finding adds knowledge to the field and opens a new range of possibilities and questions. Exploring the underlying mechanisms of the association of both school timing and baseline chronotype effects with how chronotype changes during adolescence will lead us to a better understanding of how we can help adolescents to reach healthier sleep habits.

## Methods

### Ethical approval

The study and all the methods included on it were conducted in accordance with relevant guidelines and regulations, including the ethical recommendations for human chronobiological research^100^ and Argentinian national regulations. The study was not invasive of the integrity of the participants and the data was collected during regular school hours. The study was approved by the institutional Ethical Committee of the Universidad Nacional de Quilmes (Verdict #4/2017) and by the head of school. Written informed consent was obtained from the head of the school. Students provided oral informed consent to participate.

### Participants

This study was performed in two different moments (June 2015 and July 2019) at a local secondary school in the City of Buenos Aires, Argentina (34° 60′ S, 58° 38′ W). The school year starts in March and ends in December in Buenos Aires thus, the data was collected after three/four months of classes. Those students who attended school on the corresponding day of data collection and were at 1st year (i.e. 2015) or at 5th year (i.e. 2019), were invited to participate in the study. The attendance percentage was higher than 75% on each school timing and year (2015: morning, 97.50%; afternoon, 90.24%; evening, 87.01%. 2019: morning, 75.35%; afternoon, 79.11%; evening, 91.23%) and no student refused to participate. From the 436 and 352 students who completed the questionnaire in their 1st and 5th year, respectively, 259 students were included in the analyses performed in this study. Only those students who participate on the study on both years, who maintained their original school timing and with complete data on both years were included. The final sample of students was balanced on gender (50.97% females) and it was age-homogeneous (1st year: M = 13.49 y.o., SD = 0.33; 5th year: M = 17.58 y.o., SD = 0.33).

### Procedure

A crucial aspect of our experimental setup is that in this particular school, the school timing (morning, 07:45–12:05; afternoon, 12:40–17:00; evening, 17:20–21:40) is set by a lottery system at the beginning of the secondary school and maintained during the whole secondary school, as described in depth in our previous study^57^.

Briefly, in June 2015 students in their first school year filled a questionnaire including demographic information (date of birth and self-defined gender) and the Spanish version of the MCTQ^24^. MCTQ includes questions about sleep habits and results in a local time point (MSFsc) where earlier times (i.e. low values) indicate early chronotypes and late times (i.e. high values) indicate late chronotypes^24^. Data collection was performed during students' typical school hours (morning, afternoon and evening school timings). Data collection and analysis were not performed blind to the conditions of the experiments. The exact same procedure was applied in both June 2015 (during their first school year) and July 2019 (during their last school year).

### Measurements

For each student on each school year, we obtained a chronotype index: the sleep-corrected midpoint of sleep time on free days (MSFsc)^24^, social jetlag (SJL = MSF – MSW. Note that we use the difference and not the absolute difference because we are interested in the direction of the misalignment between social and biological timings) and sleep duration on both week (SDw) and free days (SDf). From these measurements we also calculated the ΔChronotype (i.e. developmental change in chronotype, MSFsc_5th year_ – MSFsc_1st year_), the ΔSJL (i.e. SJL_5th year_ – SJL_1st year_) and the ΔSDw (i.e. SDw_5th year_ – SDw_1st year_).

Not all the variables were obtained for all students. Missing values occurred when a variable could not be calculated because of incomplete information (i.e. when a student did not complete all of the MCTQ questions). The data from a student was only included if the information was enough to calculate at least MSFsc, SJL, SDw and SDf. Missing data were omitted from the analyses.

### Statistical analysis

All statistical analyses were performed using the R system for statistical computing (v.4.0.2; R Core Team, 2020).

We ran linear mixed-effect models to determine whether school timing (morning, afternoon or evening) and age (1st or 5th school year) were associated with MSFsc or SJL. For sleep duration, the linear-mixed model included type of day of the week (weekdays or free days), school timing (morning, afternoon or evening) and age (1st or 5th school year). The same analysis was perform for total sleep duration (nocturnal sleep + naps). Students ID was included as a random effect in every model. P-values were computed using lmerTest package^101^.

We ran a generalized linear models to test whether the developmental change in chronotype depends on school timing (morning, afternoon or evening) and baseline chronotype (i.e. chronotype in 1st year) and to evaluate whether age-related changes in SJL and SDw depend on school timing (morning, afternoon or evening) and on the developmental change in chronotype.

Normality of the residuals of the models was check using Kolmogorov–Smirnov tests. Student’s t-tests were used to perform post-hoc pairwise comparisons for categorical variables. We used an alpha level of 0.05 for all of the statistical tests. When applicable, we used Bonferroni correction for multiple comparisons (corrected *P* < 0.05). Partial η^2^ effect sizes were computed *using sjstats package* version 0.18.0.

## Supplementary Information

Supplementary Information.

## Data Availability

The data and code that support the findings of this study are available from the corresponding author on request.

## References

[1] Hastings MH, Maywood ES, Brancaccio M (2018). Generation of circadian rhythms in the suprachiasmatic nucleus. Nat. Rev. Neurosci..

[2] Roenneberg T, Kumar C, Merrow M (2007). The human circadian clock entrains to sun time. Curr. Biol. CB.

[3] Wittmann M, Dinich J, Merrow M, Roenneberg T (2006). Social Jetlag: Misalignment of biological and social time. Chronobiol. Int..

[4] Casiraghi LP (2010). Human period-3 gene involvement in diurnal preference among Argentinean bipolar disorders patients. Sleep Sci..

[5] Patke A (2017). Mutation of the human circadian clock gene CRY1 in familial delayed sleep phase disorder. Cell.

[6] Hirano A (2016). A Cryptochrome 2 mutation yields advanced sleep phase in humans. ELife.

[7] Randler C, Faϐl C, Kalb N (2017). From Lark to Owl: developmental changes in morningness-eveningness from new-borns to early adulthood. Sci. Rep..

[8] Roenneberg T (2004). A marker for the end of adolescence. Curr. Biol..

[9] Kuula L (2018). Development of late circadian preference: Sleep timing from childhood to late adolescence. J. Pediatr..

[10] Karan M (2021). Sleep-wake timings in adolescence: Chronotype development and associations with adjustment. J. Youth Adolesc..

[11] Bai S, Karan M, Gonzales NA, Fuligni AJ (2021). A daily diary study of sleep chronotype among Mexican-origin adolescents and parents: Implications for adolescent behavioral health. Dev. Psychopathol..

[12] Crowley SJ, Acebo C, Carskadon MA (2007). Sleep, circadian rhythms, and delayed phase in adolescence. Sleep Med..

[13] Stothard ER (2017). Circadian entrainment to the natural light-dark cycle across seasons and the weekend. Curr. Biol..

[14] Wright KP (2013). Entrainment of the human circadian clock to the natural light-dark cycle. Curr. Biol..

[15] Leone, M. J., Sigman, M. & Golombek, D. *Effects of Social Isolation on Human Sleep and Chronotype During the COVID-19 Pandemic*. (2020) 10.2139/ssrn.3624469. 10.1016/j.cub.2020.07.015 PMC7342078 32810450

[16] Short MA (2013). A cross-cultural comparison of sleep duration between U.S. and Australian adolescents: The effect of school start time, parent-set bedtimes, and extracurricular load. Health Educ. Behav..

[17] Skeldon AC, Phillips AJK, Dijk D-J (2017). The effects of self-selected light-dark cycles and social constraints on human sleep and circadian timing: A modeling approach. Sci. Rep..

[18] Roenneberg T, Sehgal A (2015). Chapter Twelve-Human Activity and Rest In Situ. Methods in Enzymology.

[19] Santisteban JA, Brown TG, Gruber R (2018). Association between the Munich chronotype questionnaire and wrist actigraphy. Sleep Disord..

[20] Mecacci L, Zani A (1983). Morningness-eveningness preferences and sleep-waking diary data of morning and evening types in student and worker samples. Ergonomics.

[21] Duffy JF, Dijk DJ, Hall EF, Czeisler CA (1999). Relationship of endogenous circadian melatonin and temperature rhythms to self-reported preference for morning or evening activity in young and older people. J. Investig. Med. Off. Publ. Am. Fed. Clin. Res..

[22] Kantermann T, Sung H, Burgess HJ (2015). Comparing the morningness-eveningness questionnaire and Munich chronotype questionnaire to the dim light melatonin onset. J. Biol. Rhythms.

[23] Baehr EK, Revelle W, Eastman CI (2000). Individual differences in the phase and amplitude of the human circadian temperature rhythm: With an emphasis on morningness–eveningness. J. Sleep Res..

[24] Roenneberg T, Wirz-Justice A, Merrow M (2003). Life between clocks: Daily temporal patterns of human chronotypes. J. Biol. Rhythms.

[25] Horne JA, Östberg O (1976). A self-assessment questionnaire to determine morningness-eveningness in human circadian rhythms. Int. J. Chronobiol..

[26] Zavada A, Gordijn MCM, Beersma DGM, Daan S, Roenneberg T (2005). Comparison of the Munich chronotype questionnaire with the Horne-Östberg’s morningness-eveningness score. Chronobiol. Int..

[27] Fárková E, Novák JM, Manková D, Kopřivová J (2020). Comparison of Munich chronotype questionnaire (MCTQ) and morningness-eveningness questionnaire (MEQ) Czech version. Chronobiol. Int..

[28] Adan A, Natale V (2002). Gender differences in morningness-eveningness preference. Chronobiol. Int..

[29] Bailey SL, Heitkemper MM (2001). Circadian rhythmicity of cortisol and body temperature: Morningness-eveningness effects. Chronobiol. Int..

[30] Roenneberg T, Winnebeck EC, Klerman EB (2019). Daylight saving time and artificial time zones–a battle between biological and social times. Front. Physiol..

[31] Roenneberg T, Allebrandt KV, Merrow M, Vetter C (2012). Social jetlag and obesity. Curr. Biol..

[32] Parsons MJ (2015). Social jetlag, obesity and metabolic disorder: Investigation in a cohort study. Int. J. Obes..

[33] Talbot LS, McGlinchey EL, Kaplan KA, Dahl RE, Harvey AG (2010). Sleep deprivation in adolescents and adults: Changes in affect. Emotion.

[34] Levandovski R (2011). Depression scores associate with chronotype and social jetlag in a rural population. Chronobiol. Int..

[35] Carskadon MA (2002). Adolescent Sleep Patterns: Biological, Social, and Psychological Influences.

[36] McKnight-Eily LR (2011). Relationships between hours of sleep and health-risk behaviors in US adolescent students. Prev. Med..

[37] Díaz-Morales JF, Escribano C (2015). Social jetlag, academic achievement and cognitive performance: Understanding gender/sex differences. Chronobiol. Int..

[38] Mak K-K, Lee S-L, Ho S-Y, Lo W-S, Lam T-H (2012). Sleep and Academic performance in Hong Kong adolescents. J. Sch. Health.

[39] Carskadon MA (2011). Sleep in adolescents: The perfect storm. Pediatr. Clin. North Am..

[40] Owens JA, Belon K, Moss P (2010). Impact of delaying school start time on adolescent sleep, mood, and behavior. Arch. Pediatr. Adolesc. Med..

[41] Dunster GP (2018). Sleepmore in Seattle: Later school start times are associated with more sleep and better performance in high school students. Sci. Adv..

[42] Kelley P, Lockley SW, Kelley J, Evans MDR (2017). Is 8:30 a.m. still too early to start school? A 10:00 a.m. school start time improves health and performance of students aged 13–16. Front. Hum. Neurosci..

[43] Lo JC (2018). Sustained benefits of delaying school start time on adolescent sleep and well-being. Sleep.

[44] Boergers J, Gable CJ, Owens JA (2014). Later school start time is associated with improved sleep and daytime functioning in adolescents. J. Dev. Behav. Pediatr..

[45] Wahlstrom K (2010). School start time and sleepy teens. Arch. Pediatr. Adolesc. Med..

[46] Wheaton AG, Chapman DP, Croft JB (2016). School start times, sleep, behavioral, health, and academic outcomes: A review of the literature. J. Sch. Health.

[47] Vorona RD (2011). Dissimilar teen crash rates in two neighboring Southeastern Virginia cities with different high school start times. J. Clin. Sleep Med..

[48] Borlase BJ, Gander PH, Gibson RH (2013). Effects of school start times and technology use on teenagers’ sleep: 1999–2008. Sleep Biol. Rhythms.

[49] Carskadon MA, Wolfson AR, Acebo C, Tzischinsky O, Seifer R (1998). Adolescent sleep patterns, circadian timing, and sleepiness at a transition to early school days. Sleep.

[50] Thacher PV, Onyper SV (2016). Longitudinal outcomes of start time delay on sleep, behavior, and achievement in high school. Sleep.

[51] Owens JA, Dearth-Wesley T, Herman AN, Oakes JM, Whitaker RC (2017). A quasi-experimental study of the impact of school start time changes on adolescent sleep. Sleep Health.

[52] Escribano C, Díaz-Morales JF (2014). Daily fluctuations in attention at school considering starting time and chronotype: An exploratory study. Chronobiol. Int..

[53] Estevan I, Silva A, Tassino B (2018). School start times matter, eveningness does not. Chronobiol. Int..

[54] Estevan I, Silva A, Vetter C, Tassino B (2020). Short sleep duration and extremely delayed chronotypes in uruguayan youth: The role of school start times and social constraints. J. Biol. Rhythms.

[55] Arrona-Palacios A, Díaz-Morales JF (2018). Morningness–eveningness is not associated with academic performance in the afternoon school shift: Preliminary findings. Br. J. Educ. Psychol..

[56] Carvalho-Mendes RP, Dunster GP, de la Iglesia HO, Menna-Barreto L (2020). Afternoon school start times are associated with a lack of both social jetlag and sleep deprivation in adolescents. J. Biol. Rhythms.

[57] Goldin AP, Sigman M, Braier G, Golombek DA, Leone MJ (2020). Interplay of chronotype and school timing predicts school performance. Nat. Hum. Behav..

[58] Vollmer C, Schaal S, Hummel E, Randler C (2011). Association among school-related, parental and self-related problems and morningness–eveningness in adolescents. Stress Health.

[59] Carskadon MA, Vieira C, Acebo C (1993). Association between puberty and delayed phase preference. Sleep.

[60] Hagenauer MH, Ku JH, Lee TM (2011). Chronotype changes during puberty depend on gonadal hormones in the slow-developing rodent, Octodon degus. Horm. Behav..

[61] Reinberg A, Ashkenazi I (2008). Internal desynchronization of circadian rhythms and tolerance to shift work. Chronobiol. Int..

[62] Roenneberg T, Hut R, Daan S, Merrow M (2010). Entrainment concepts revisited. J. Biol. Rhythms.

[63] Sack RL, Blood ML, Lewy AJ (1992). Melatonin rhythms in night shift workers. Sleep.

[64] Zerbini G, Merrow M (2017). Time to learn: How chronotype impacts education. PsyCh J..

[65] Preckel F (2013). Morningness-eveningness and educational outcomes: The lark has an advantage over the owl at high school. Br. J. Educ. Psychol..

[66] Dewald JF, Meijer AM, Oort FJ, Kerkhof GA, Bögels SM (2010). The influence of sleep quality, sleep duration and sleepiness on school performance in children and adolescents: A meta-analytic review. Sleep Med. Rev..

[67] Santhi N (2013). Morning sleep inertia in alertness and performance: Effect of cognitive domain and white light conditions. PLOS ONE.

[68] Haraszti RÁ, Ella K, Gyöngyösi N, Roenneberg T, Káldi K (2014). Social jetlag negatively correlates with academic performance in undergraduates. Chronobiol. Int..

[69] Touitou Y (2013). Adolescent sleep misalignment: A chronic jet lag and a matter of public health. J. Physiol. Paris.

[70] Jankowski KS (2017). Social jet lag: Sleep-corrected formula. Chronobiol. Int..

[71] Shalini P (2021). Recommended amount of sleep for pediatric populations: A consensus statement of the American Academy of Sleep Medicine. J. Clin. Sleep Med..

[72] Watson NF (2021). Delaying middle school and high school start times promotes student health and performance: An American Academy of sleep medicine position statement. J. Clin. Sleep Med..

[73] Hirshkowitz M (2015). National sleep foundation’s updated sleep duration recommendations: Final report. Sleep Health.

[74] Martínez-Lozano N (2020). Evening types have social jet lag and metabolic alterations in school-age children. Sci. Rep..

[75] Tzischinsky O, Shochat T (2011). Eveningness, sleep patterns, daytime functioning, and quality of life in israeli adolescents. Chronobiol. Int..

[76] Vitale JA (2015). Chronotype influences activity circadian rhythm and sleep: Differences in sleep quality between weekdays and weekend. Chronobiol. Int..

[77] Adan A (2012). Circadian typology: A comprehensive review. Chronobiol. Int..

[78] Roenneberg T (2015). Having trouble typing? What on earth is chronotype?. J. Biol. Rhythms.

[79] Roenneberg T, Pilz LK, Zerbini G, Winnebeck EC (2019). Chronotype and social jetlag: A (Self-) critical review. Biology.

[80] Hagenauer MH, Lee TM (2012). The neuroendocrine control of the circadian system: Adolescent chronotype. Front. Neuroendocrinol..

[81] Reinberg A (1988). Alteration of period and amplitude of circadian rhythms in shift workers. Eur. J. Appl. Physiol..

[82] van der Vinne V (2015). Timing of examinations affects school performance differently in early and late chronotypes. J. Biol. Rhythms.

[83] Carissimi A (2016). The influence of school time on sleep patterns of children and adolescents. Sleep Med..

[84] Bland JM, Altman DG (1994). Statistics notes: Some examples of regression towards the mean. BMJ.

[85] Borisenkov MF, Perminova EV, Kosova AL (2010). Chronotype, sleep length, and school achievement of 11- to 23-year-old students in northern European Russia. Chronobiol. Int..

[86] Jankowski KS (2015). Composite scale of morningness: Psychometric properties, validity with Munich chronotype questionnaire and age/sex differences in Poland. Eur. Psychiatry.

[87] Carskadon MA, Wolfson AR, Acebo C, Tzischinsky O, Seifer R (1998). Adolescent sleep patterns, circadian timing, and sleepiness at a transition to early school days. Sleep.

[88] Russo PM, Bruni O, Lucidi F, Ferri R, Violani C (2007). Sleep habits and circadian preference in Italian children and adolescents. J. Sleep Res..

[89] Cheung FTW (2022). Validation of the Chinese version of the Munich chronotype questionnaire (MCTQHK) in Hong Kong Chinese youths. Chronobiol. Int..

[90] Saxvig IW (2021). Sleep during COVID-19-related school lockdown, a longitudinal study among high school students. J. Sleep Res..

[91] Arrona-Palacios A, García A, Valdez P (2015). Sleep–wake habits and circadian preference in Mexican secondary school. Sleep Med..

[92] Martin JS, Gaudreault MM, Perron M, Laberge L (2016). Chronotype, light exposure, sleep, and daytime functioning in high school students attending morning or afternoon school shifts: An actigraphic study. J. Biol. Rhythms.

[93] Randler C (2008). Morningness-eveningness comparison in adolescents from different countries around the world. Chronobiol. Int..

[94] Arora T, Taheri S (2015). Associations among late chronotype, body mass index and dietary behaviors in young adolescents. Int. J. Obes..

[95] Borisenkov MF, Polugrudov AS, Paderin NM, Bakutova LA (2019). Young inhabitants of the North with late chronotype and social jetlag consume more high-calorie foods and alcohol. Biol. Rhythm Res..

[96] Haynie DL (2018). Beyond sleep duration: Bidirectional associations among chronotype, social jetlag, and drinking behaviors in a longitudinal sample of US high school students. Sleep.

[97] Malone SK (2016). Characteristics associated with sleep duration, chronotype, and social jet lag in adolescents. J. Sch. Nurs..

[98] Schlarb AA, Sopp R, Ambiel D, Grünwald J (2014). Chronotype-related differences in childhood and adolescent aggression and antisocial behavior–A review of the literature. Chronobiol. Int..

[99] Taylor BJ, Hasler BP (2018). Chronotype and mental health: Recent advances. Curr. Psychiatry Rep..

[100] Portaluppi F, Smolensky MH, Touitou Y (2010). Ethics and methods for biological rhythm research on animals and human beings. Chronobiol. Int..

[101] Kuznetsova A, Brockhoff PB, Christensen RHB (2017). lmerTest package: Tests in linear mixed effects models. J. Stat. Softw..

