# Supplementary material for: Chronotype at the beginning of secondary school and school timing are both associated with chronotype development during adolescence
Source: Sci Rep. 2022 May 17;12:8207. doi: 10.1038/s41598-022-11928-9 (PMC9114414; doi:10.1038/s41598-022-11928-9)
Supplement: Supplementary file 1 — Supplementary Information. [file 41598_2022_11928_MOESM1_ESM.docx]

**Supplementary Information**

**Supplementary Figures**

**
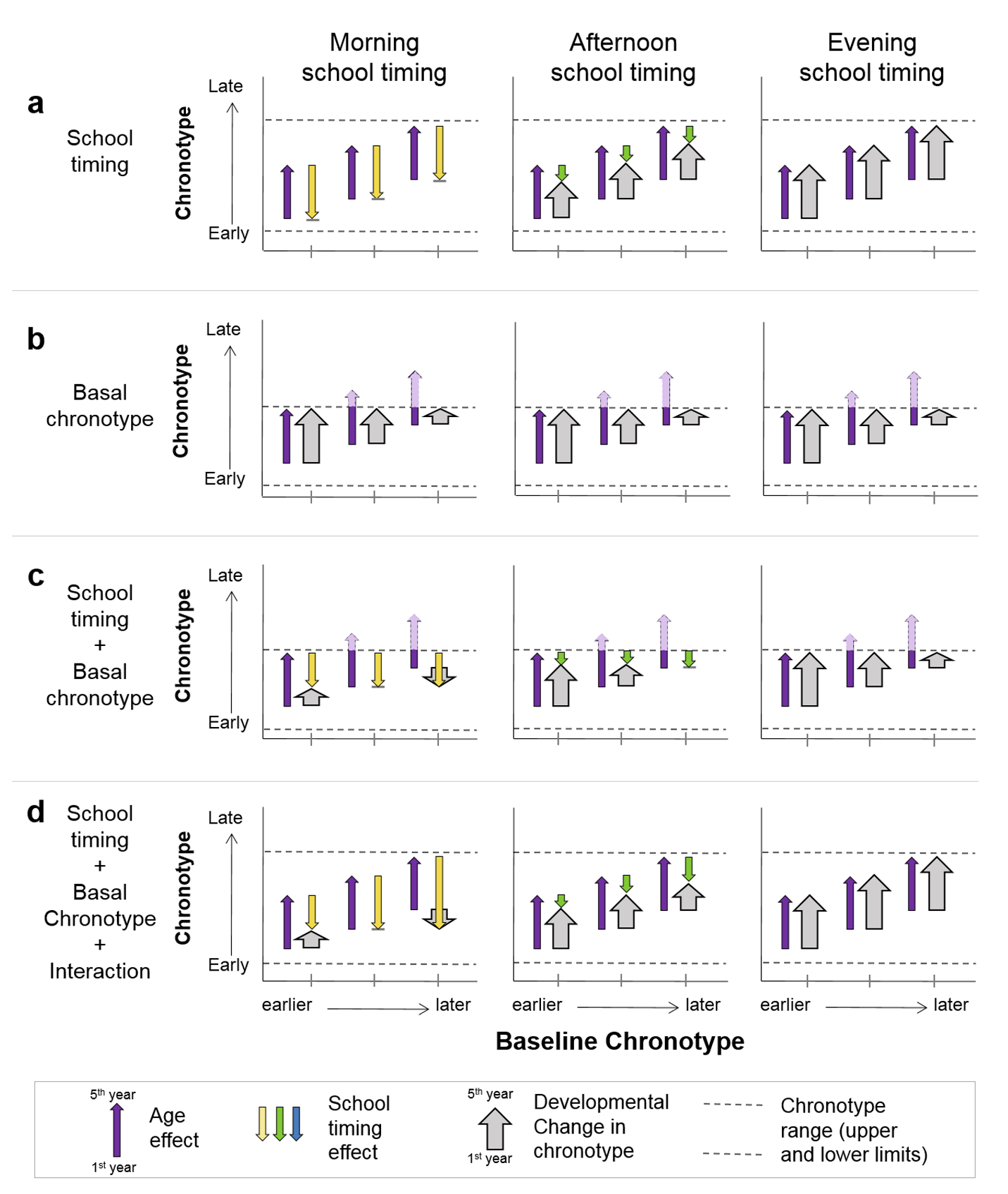
**

**Supplementary Figure 1. Mechanisms underlying the theoretical scenarios of the effect of school timing and baseline chronotype (in 1^st^ year of secondary school) on ΔChronotype (MSFsc 5^th^ year – MSFsc 1^st^ year) according to four different hypotheses.** The graphs include plots of the Chronotype as a function of baseline chronotype, where the base of wide arrows represent students’ chronotype in their 1^st^ year (i.e. the baseline chronotype) and its arrowheads represent students’ chronotype in their 5^th^ year. Dark violet narrow arrows represent the expected age-related change in chronotype, narrow colored inverted arrows (yellow, green and blue) represent the expected effect of each school timing on students’ chronotype (morning, afternoon and evening, respectively). Note that the sum of corresponding dark violet and the school timing inverted narrow arrows results in the gray wide arrow, which represents ΔChronotype. That is why when the dark violet arrow is equal to the colored inverted arrow, there is not a wide gray arrow. The horizontal dotted lines delimit the mean range of stable chronotypes. As a theoretical range, the exact values of limits are unknown. Thus, different positions of these limits in conjunction with school timing are associated with different expected effects and, consistently, different scenarios with different effects on ΔChronotype. In particular, in scenarios (a) and (d), the limit does not affect ΔChronotype, but in scenarios (b) and (c) it does. If the expected age-related change in chronotype exceeds the limits of synchronization, the corresponding dark violet arrows turns into light violet arrows when crossing the upper limit, which represents that the extra chronotype delay will not be effective to achieve the proper synchronization. **a- Only school timing has an effect.** As a social cue, school timing influences adolescents’ late chronotypes. Specifically, an earlier school timing exerts more pressure than a later school timing and it will be associated with a lower ΔChronotype. The pressure exerted by school timing does not depend on the baseline chronotype and, as a consequence, all baseline chronotypes are equally affected by each school timing. As a result, the slopes of the linear relationship between ΔChronotype and baseline chronotype will be zero, with increasingly higher intercepts as the school timing gets later. **b- Only the baseline chronotype has an effect.** As the range of possible chronotypes is limited, if the expected age-related change on chronotype exceeds these limits, the observed ΔChronotype would be smaller than expected. As adolescence is associated with a progressive delay in chronotype, we hypothesize that students with later baseline chronotypes will reach the aforementioned upper limit faster than students with earlier chronotypes. This situation leads to a larger change in chronotype for earlier chronotypes, resulting in a similar and negative slope without differences between school timings (neither on slope nor on the intercept). **C- Baseline chronotype and school timing have additive effects.** This is a combination of the previously described two scenarios: ΔChronotype is independently affected by both baseline chronotype and school timing. Similar, and negative, slopes will be expected for all school timings (i.e. baseline chronotype effect) but increasingly higher intercepts (i.e. school timing effect) as school timing gets later. **d- Baseline chronotype and school timing interaction.** This last scenario might only be possible if the expected age- and school timing changes are within the range of possible stable chronotypes. In this scenario, the magnitude of the effect of school timing will be higher when the baseline chronotype is later for morning and afternoon school timings, while the evening school timing will be late enough not to exert pressure, regardless of baseline chronotype.


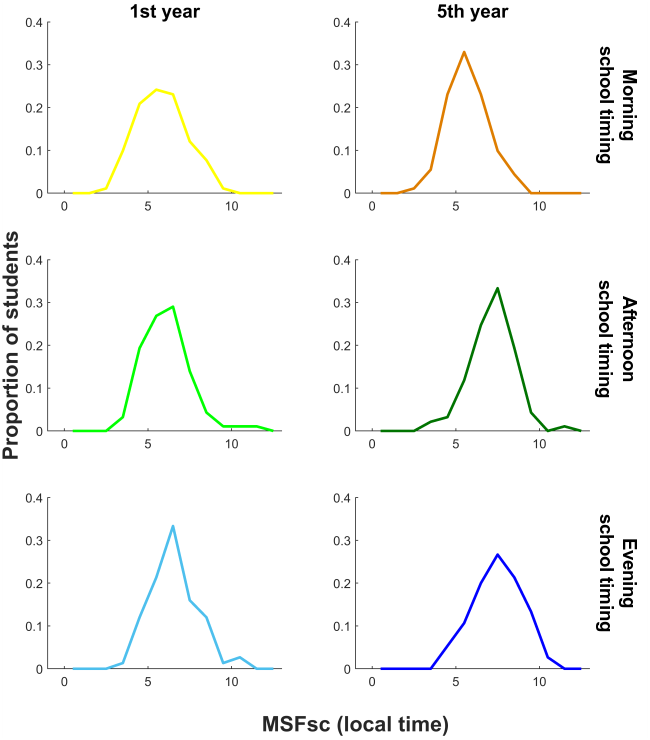


**Supplementary Figure 2. Chronotype distributions for all students according to their age and school timing.** The midpoint of sleep on free days, sleep corrected (MSFsc) is depicted. Left column: MSFsc distribution for 1^st^ year students. Right column: MSFsc distribution for 5^th^ year students. Rows 1-3: Morning, Afternoon and Evening, respectively. N=259 (the same group of students on both 1^st^ and 5^th^ year). All values are represented in hours (local time, h).


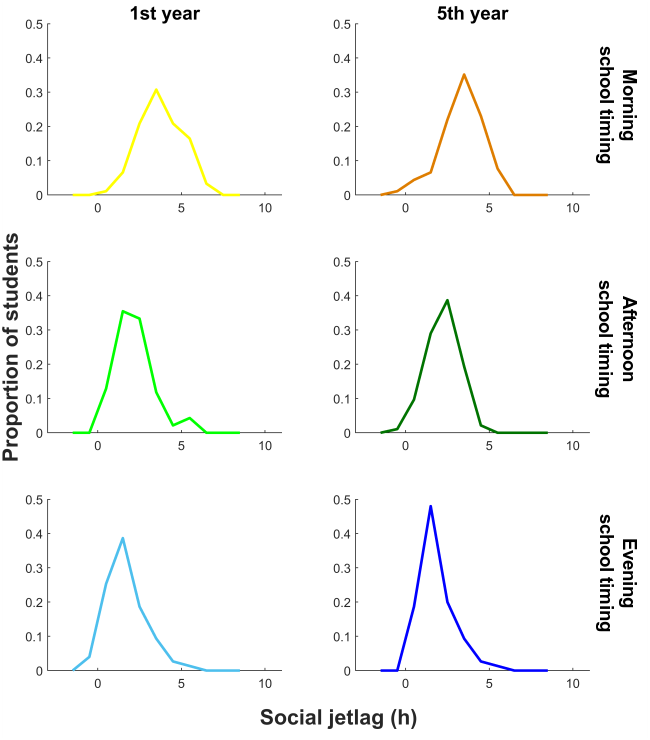


**Supplementary Figure 3. Social jetlag distributions for all students according to their age and school timing.** Left column: SJL distribution for 1^st^ year students. Right column: SJL distribution for 5^th^ year students. Rows 1-3: Morning, Afternoon and Evening, respectively. N= 259 (the same group of students on both 1^st^ and 5^th^ year). SJL values are represented in hours (h).


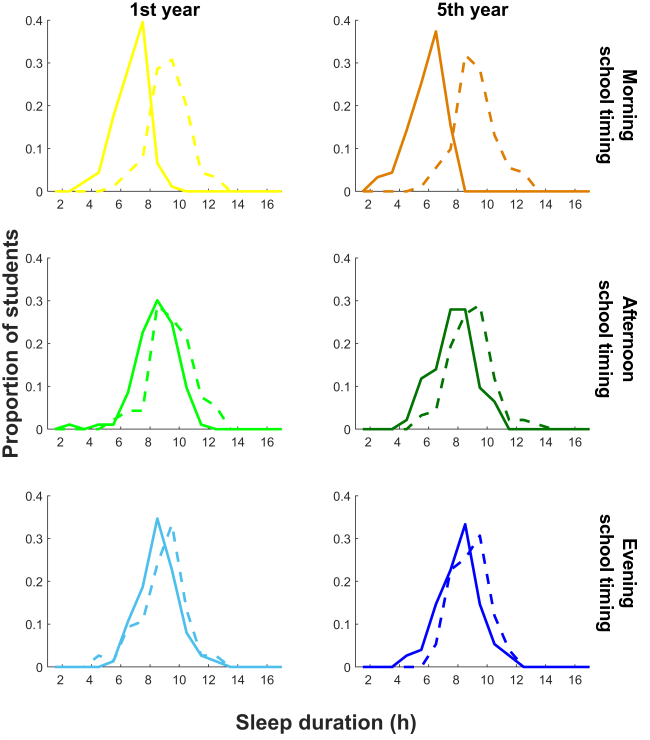


**Supplementary Figure 4. Sleep duration distributions for all students according to their age and school timing.** The sleep duration on weekdays (full lines) and on free days (dashed lines) is depicted. Left column: sleep duration distribution for 1^st^ year students. Right column: sleep duration distribution for 5^th^ year students. Rows 1-3: Morning, Afternoon and Evening, respectively. N= 259 (the same group of students on both 1^st^ and 5^th^ year). Sleep duration values are represented in hours (h).


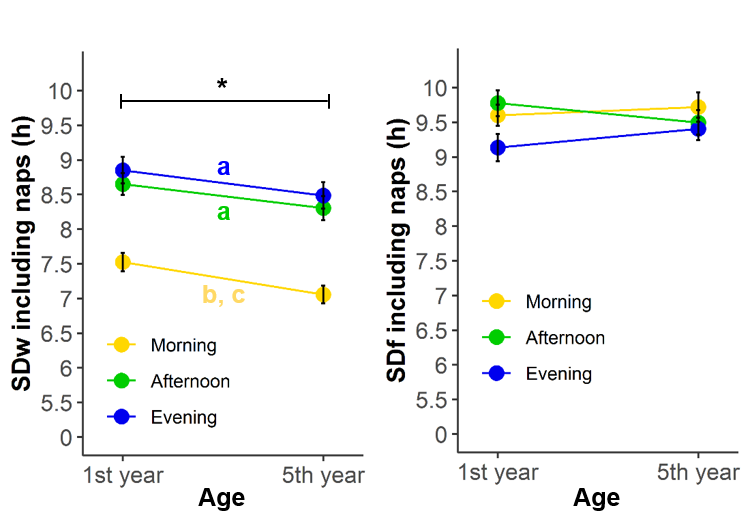


**Supplementary Figure 5. Naps only partially compensate for the short sleep duration on weekdays (SDw). a- Adolescents’ total sleep duration on weekdays (SDw + nap time, tSDw) depends on school timing and age.** Students sleep less when they are older and when attending the morning school timing, even considering naps. tSDw does not depend on the interaction between school timing and age, that is, adolescents sleep less in their 5^th^ year regardless of their school timing. **b- Total sleep duration in free days (tSDf) does not depend on school timing nor age.** Students sleep more on free days than on weekdays, even including naps, independently of their age and school timing. No differences were found between school timings and school year on free days. Post-hoc comparisons, p<.0038 (p<.05, Bonferroni corrected). Data are M ± SEM; n = 257. Note that the assumption of normality of residual of this model was rejected (p=0.037). Lowercase letters indicate significant differences between groups: a, compared with morning; b, compared with afternoon; c, compared with evening. *: significant difference between 1^st^ and 5^th^ year without differentiate between school timings.

**
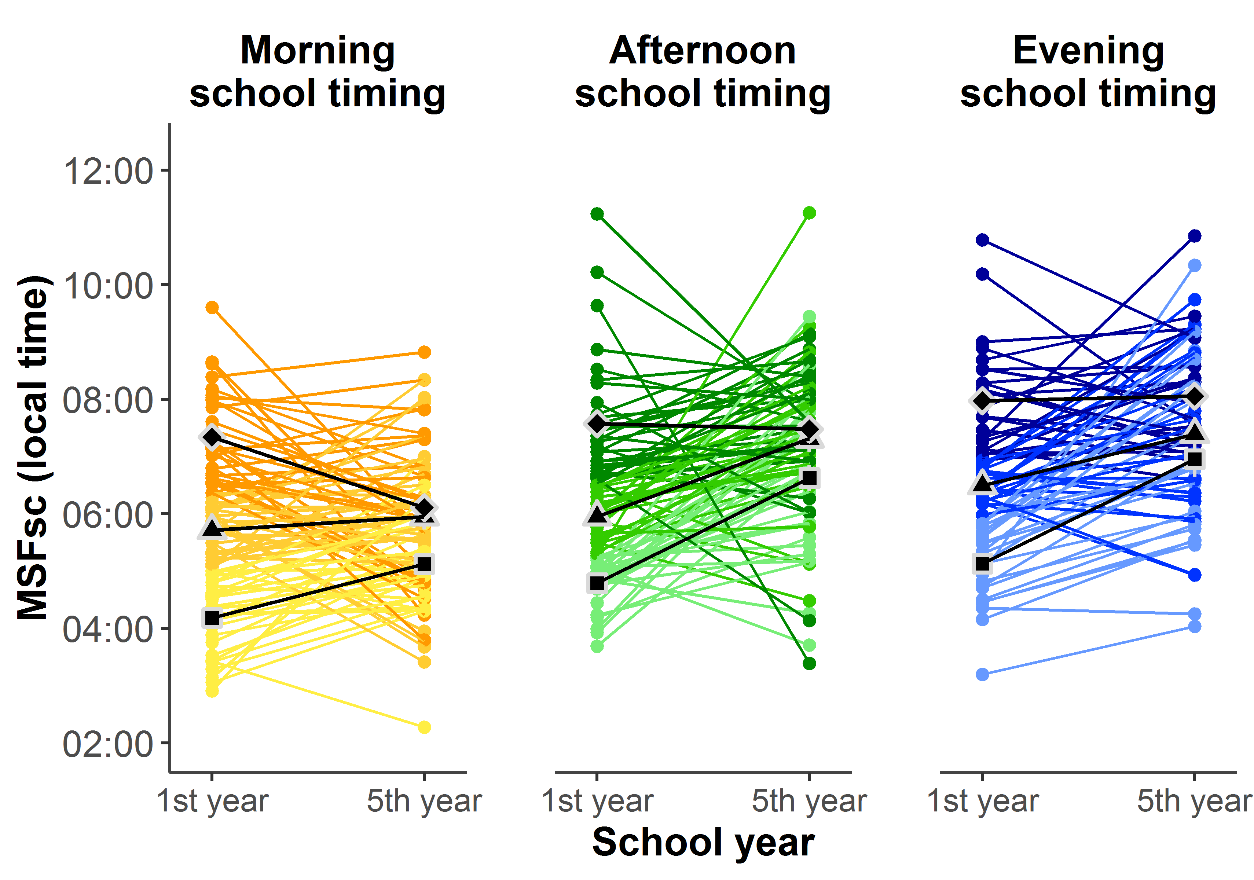
**

**Supplementary Figure 6. Change in individual chronotypes during adolescence.** Color intensity indicates MSFsc tertile in 1^st^ year, with darker colors for later baseline chronotypes tertiles. Black shapes represent the MSFsc mean for each year and tertile (☐: early tertile, △: intermediate tertile and ◇: late tertile).

**
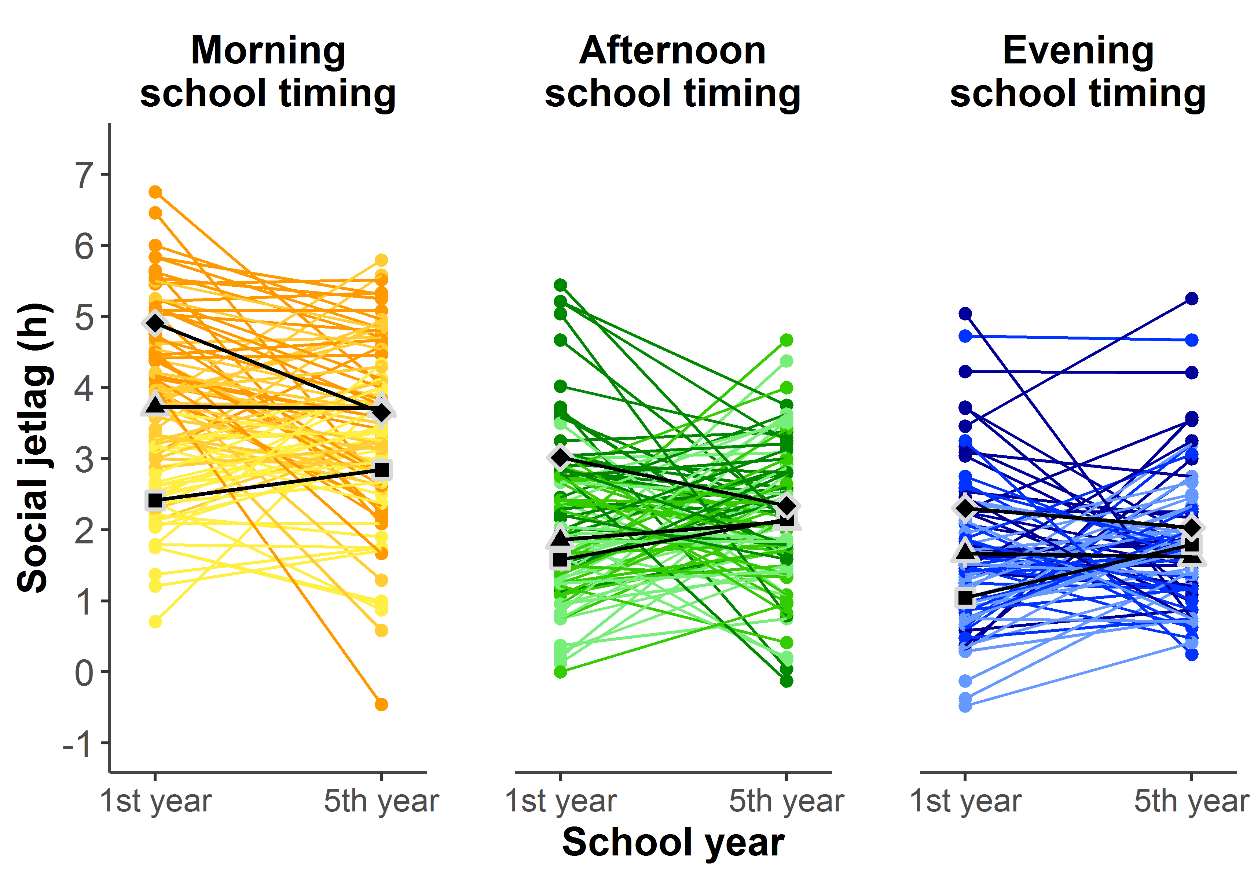
**

**Supplementary Figure 7. Change in individual Social jetlag during adolescence.** Color intensity indicates MSFsc tertile in 1^st^ year, with darker colors for later baseline chronotypes tertiles. Black shapes represent the MSFsc mean for each year and tertile (☐: early tertile, △: intermediate tertile and ◇: late tertile).


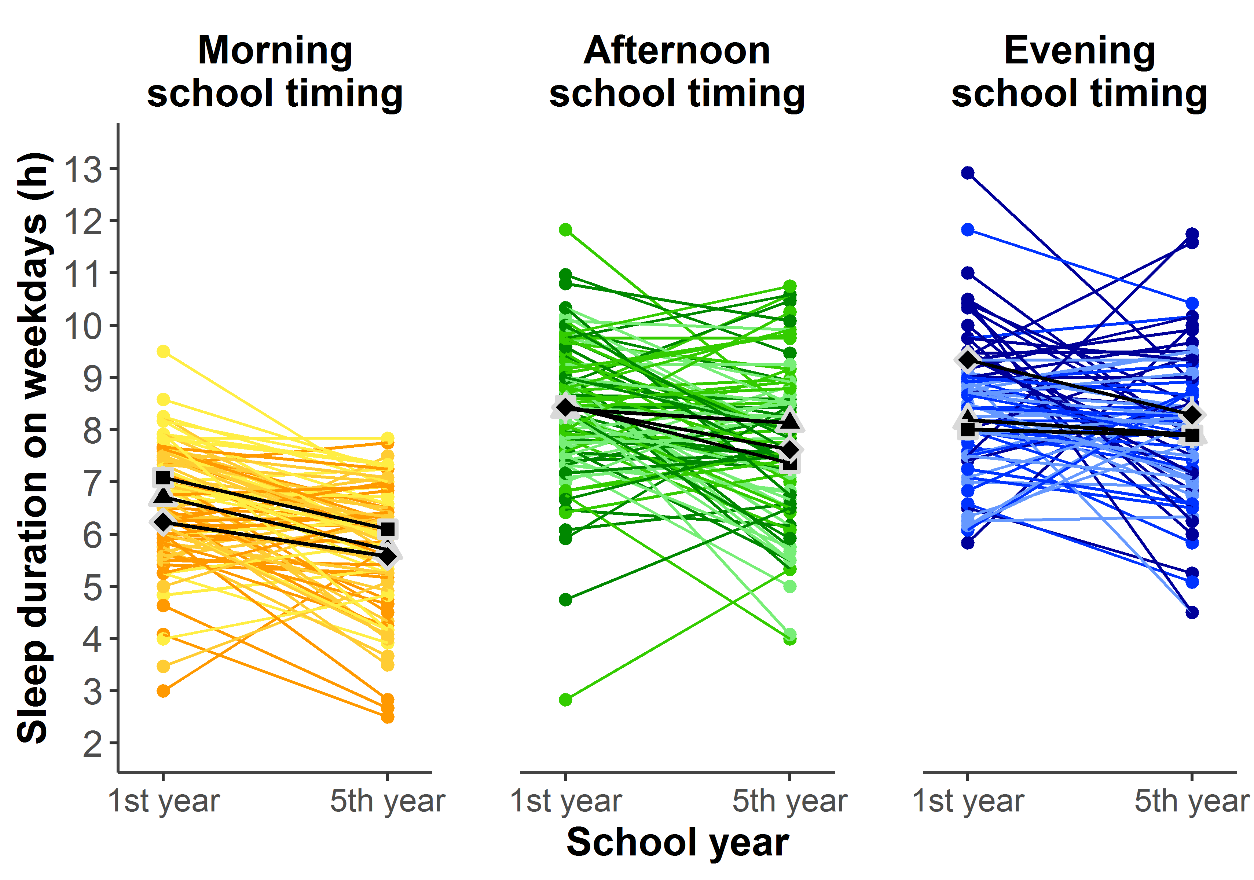


**Supplementary Figure 8. Change in individual Sleep duration during adolescence.** Color intensity indicates MSFsc tertile in 1^st^ year, with darker colors for later baseline chronotypes tertiles. Black shapes represent the MSFsc mean for each year and tertile (☐: early tertile, △: intermediate tertile and ◇: late tertile).

**Supplementary Tables**

**Supplementary Table 1. ANOVA results for MSFsc model (MSFsc, Figure 1.a).**

|  | **Sum Sq** | **d.f** | **F** | **P** | **Partial η2** | **90% CI** |
| --- | --- | --- | --- | --- | --- | --- |
| School year | 52.077 | 1 | 41.921 | <0.0001 | 0.141 | [0.081 to 0.207] |
| School timing | 73.783 | 2 | 29.697 | <0.0001 | 0.188 | [0.119 to 0.256] |
| School year:School timing | 29.968 | 2 | 12.062 | <0.0001 | 0.086 | [0.036 to 0.142] |

**Supplementary Table 2. Summary results for the model including MSFsc model (MSFsc, Figure 1a).**

|  |  | **Beta** | **95% CI** | **t-value** | **p-value** |
| --- | --- | --- | --- | --- | --- |
| 0 | Intercept | 5.762 | [5.485 to 6.039] | 40.648 | <0.0001 |
| 1 | 5th year | -0.292 | [-0.352 to 0.294] | -0.176 | 0.860 |
| 2 | Afternoon | 0.361 | [-0.029 to 0.750] | 1.809 | 0.071 |
| 3 | Evening | 0.790 | [0.379 to 1.202] | 3.748 | <0.001 |
| 4 | 5th year:Afternoon | 1.047 | [0.592 to 1.502] | 4.505 | <0.0001 |
| 5 | 5th year:Evening | 0.952 | [0.471 to 1.433] | 3.872 | <0.001 |

**Supplementary Table 3. Post-hoc comparisons for Midpoint of sleep on free days, sleep corrected (MSFsc, Figure 1a). Post-hoc pairwise [(1) – (2)] comparisons were significant at p<.006 (p<.05, Bonferroni corrected).**

| **Age**  **group** | **SchoolTiming**  **(1) (2)** | | **t-value** | **df** | **p value** | **Cohen's d** | **95% CI** |  |
| --- | --- | --- | --- | --- | --- | --- | --- | --- |
| 1st year | Morning | Afternoon | -1.809 | 464 | 0.641 | -0.324 | [-0.676 to 0.029] | |
| 1st year | Morning | Evening | -3.748 | 464 | 0.002 | -0.709 | [-1.083 to -0.335] | |
| 1st year | Afternoon | Evening | -2.048 | 464 | 0.370 | -0.386 | [-0.756 to -0.015] | |
| 5th year | Morning | Afternoon | -7.060 | 464 | <0.0001 | -1.263 | [-1.623 to -0.903] | |
| 5th year | Morning | Evening | -8.260 | 464 | <0.0001 | -1.563 | [-1.947 to -1.179] | |
| 5th year | Afternoon | Evening | -1.593 | 464 | 1 | -0.300 | [-0.671 to 0.070] | |
|  |  |  |  |  |  |  |  | |
| **School**  **Timing** | **Agegroup**  **(1) (2)** | |  |  |  |  |  |  |
| Morning | 1st year | 5th year | 0.176 | 256 | 1 | 0.026 | [-0.265 to 0.317] | |
| Afternoon | 1st year | 5th year | -6.228 | 256 | <0.0001 | -0.913 | [-1.207 to -0.620] | |
| Evening | 1st year | 5th year | -5.069 | 256 | <0.0001 | -0.828 | [-1.153 to -0.503] | |

**Supplementary Table 4. ANOVA results for Social jetlag model (SJL, Figure 1b).**

|  | **Sum Sq** | **d.f** | **F** | **P** | **Partial η2** | **90% CI** |
| --- | --- | --- | --- | --- | --- | --- |
| School year | 0.187 | 1 | 0.194 | 0.660 | 0.001 | [0.000 to 0.016] |
| School timing | 188.655 | 2 | 97.691 | <0.0001 | 0.433 | [0.360 to 0.496] |
| School year:School timing | 4.418 | 2 | 2.288 | 0.104 | 0.018 | [0.000 to 0.048] |

**Supplementary Table 5. Summary results for Social jetlag model (SJL, Figure 1b).**

|  |  | **Beta** | **95% CI** | **t-value** | **p-value** |
| --- | --- | --- | --- | --- | --- |
| 0 | Intercept | 3.696 | [3.467 to 3.926] | 31.433 | <0.0001 |
| 1 | 5th year | -0.293 | [-0.578 to -0.008] | -2.013 | 0.045 |
| 2 | Afternoon | -1.532 | [-1.855 to -1.209] | -9.261 | <0.0001 |
| 3 | Evening | -2.018 | [-2.359 to -1.676] | -11.534 | <0.0001 |
| 4 | 5th year:Afternoon | 0.331 | [-0.069 to 0.732] | 1.617 | 0.107 |
| 5 | 5th year:Evening | 0.434 | [0.010 to 0.858] | 2.002 | 0.046 |

**Supplementary Table 6. Post-hoc comparisons for Social jetlag (SJL, Figure 1.b). Post-hoc pairwise [(1) – (2)] comparisons were significant at p<.017 (p<.05, Bonferroni corrected).**

| **School Timing**   1. **(2)** | | **t-value** | **df** | **p value** | **Cohen's d** | **95% CI** |  |
| --- | --- | --- | --- | --- | --- | --- | --- |
| Morning | Afternoon | 10.520 | 256 | <0.0001 | 1.390 | [1.116 to 1.664] |  |
| Morning | Evening | 13.112 | 256 | <0.0001 | 1.833 | [1.535 to 2.130] |  |
| Afternoon | Evening | 3.182 | 256 | 0.005 | 0.443 | [0.167 to 0.718] |  |

**Supplementary Table 7. Sleep onset and offset (wake-up time) on weekdays and free days for adolescents attending school on different school timings at their 1^st^ and 5^th^ secondary school year. Values represent local times (hh:mm, M±SD).**

| School Timing | Weekdays | | | | | |
| --- | --- | --- | --- | --- | --- | --- |
|  | 1st year | | 5th year | | | |
|  | Sleep offset  (Wake-up) | Sleep onset | Sleep offset  (Wake-up) | Sleep onset | | |
| Morning | 06:19 ± 00:22 | 23:39 ± 01:09 | 06:25 ± 00:30 | 00:38 ± 01:14 | | |
| Afternoon | 08:31 ± 01:02 | 00:05 ± 01:08 | 09:12 ± 01:16 | 01:30 ± 01:13 | | |
| Evening | 09:15 ± 01:21 | 00:44 ± 00:92 | 09:58 ± 01:25 | 01:55 ± 01:13 | | |
|  |  |  |  |  |  |  |
|  |  |  |  |  |  |  |
| School Timing | Free days | | | | | |
|  | 1st year | | 5th year | | | |
|  | Sleep offset  (Wake-up) | Sleep onset | Sleep offset  (Wake-up) | Sleep onset | | |
| Morning | 11:18 ± 01:47 | 02:04 ± 01:43 | 11:30 ± 01:41 | 02:22 ± 01:23 | | |
| Afternoon | 11:10 ± 01:32 | 01:46 ± 01:29 | 11:59 ± 01:31 | 03:08 ± 01:20 | | |
| Evening | 11:05 ± 01:37 | 02:15 ± 01:16 | 12:11 ± 01:32 | 03:21 ± 01:25 | | |

**Supplementary Table 8. ANOVA results for Sleep Duration model (SD, Figures 1c).**

|  | **Sum Sq** | **d.f** | **F** | **P** | **Partial η2** | **90% CI** |
| --- | --- | --- | --- | --- | --- | --- |
| School year | 53.521 | 1 | 35.032 | <0.0001 | 0.044 | [0.023 to 0.070] |
| School timing | 97.957 | 2 | 32.059 | <0.0001 | 0.200 | [0130 to 0.313] |
| Type of day | 599.281 | 1 | 392.264 | <0.0001 | 0.338 | [0.276 to 0.379] |
| School year:School timing | 6.104 | 2 | 1.998 | 0.1363 | 0.005 | [0.000 to 0.015] |
| School year:Type of day | 15.456 | 1 | 10.117 | 0.0016 | 0.013 | [0.003 to 0.029] |
| School timing:Type of day | 275.489 | 2 | 90.161 | <0.0001 | 0.190 | [0.150 to 0.230] |
| School year:School timing:Type of day | 4.180 | 2 | 1.368 | 0.2552 | 0.004 | [0.000 to 0.012] |

**Supplementary Table 9. Summary results for Sleep Duration model (SD, Figures 1c).**

|  |  | **Beta** | **95% CI** | **t-value** | **p-value** |
| --- | --- | --- | --- | --- | --- |
| 0 | Intercept | 9.231 | [8.954 to 9.509] | 64.801 | <0.0001 |
| 1 | 5th year | -0.086 | [-0.444 to 0.271] | -0.471 | 0.638 |
| 2 | Afternoon | 0.169 | [-0.222 to 0.560] | 0.842 | 0.400 |
| 3 | Evening | -0.386 | [-0.800 to 0.027] | -1.823 | 0.069 |
| 4 | Weekdays | -2.560 | [-2.917 to -2.202] | -13.969 | <0.0001 |
| 5 | 5th year:Afternoon | -0.450 | [0.953 to 0.052] | -1.748 | 0.081 |
| 6 | 5th year:Evening | 0.075 | [-0.456 to 0.607] | 0.276 | 0.782 |
| 7 | 5th year:Weekdays | -0.797 | [-1.303 to -0.292] | -3.077 | 0.002 |
| 8 | Afternoon:Weekdays | 1.590 | [1.087 to 2.092] | 6.167 | <0.0001 |
| 9 | Evening:Weekdays | 2.241 | [1.709 to 2.773] | 8.221 | <0.0001 |
| 10 | 5th year:Afternoon:Weekdays | 0.603 | [-0.108 to 1.313] | 1.654 | 0.099 |
| 11 | 5th year:Evening:Weekdays | 0.316 | [-0.436 to 1.069] | 0.821 | 0.412 |

**Supplementary Table 10. Post-hoc comparisons for Sleep duration (SD, Figure 1c). Post-hoc pairwise [(1) – (2)] comparisons were significant at p<.0038 (p<.05, Bonferroni corrected).**

| **Day of the week** | **School timing (1) (2)** | | **t-value** | **df** | **p value** | **Cohen's d** | **95% CI** |
| --- | --- | --- | --- | --- | --- | --- | --- |
| Free days | Morning | Afternoon | 0.368 | 555 | 1 | 0.046 | [-0.198 to 0.290] |
| Free days | Morning | Evening | 2.149 | 555 | 0.417 | 0.282 | [0.024 to 0.540] |
| Free days | Afternoon | Evening | 1.809 | 555 | 0.922 | 0.236 | [-0.020 to 0.493] |
| Weekdays | Morning | Afternoon | -11.956 | 555 | <0.0001 | -1.484 | [-1.736 to -1.232] |
| Weekdays | Morning | Evening | -12.635 | 555 | <0.0001 | -1.659 | [-1.927 to -1.391] |
| Weekdays | Afternoon | Evening | -1.337 | 555 | 1 | -0.175 | [-0.432 to 0.082] |
|  |  |  |  |  |  |  |  |
| **Day of the week** | **Age group (1) (2)** | |  |  |  |  |  |
| Free days | 1st year | 5th year | 1.936 | 768 | 0.692 | 0.171 | [-0.002 to 0.344] |
| Weekdays | 1st year | 5th year | 6.434 | 768 | <0.0001 | 0.568 | [0.393 to 0.743] |
|  |  |  |  |  |  |  |  |
| **School  Timing** | **Day of the week (1) (2)** | |  |  |  |  |  |
| Morning | Free days | Weekdays | 22.832 | 768 | <0.0001 | 2.393 | [2.163 to 2.624] |
| Afternoon | Free days | Weekdays | 8.327 | 768 | <0.0001 | 0.863 | [0.656 to 1.071] |
| Evening | Free days | Weekdays | 3.917 | 768 | 0.001 | 0.452 | [0.225 to 0.680] |
|  |  |  |  |  |  |  |  |
| **Age group** | **Day of the week (1) (2)** | |  |  |  |  |  |
| 1st year | Free days | Weekdays | 11.756 | 768 | <0.0001 | 1.038 | [0.859 to 1.217] |
| 5th year | Free days | Weekdays | 16.254 | 768 | <0.0001 | 1.435 | [1.251 to 1.619] |

**Supplementary Table 11. ANOVA results for Total Sleep Duration model (tSD, Supp. Figure 5).**

|  | **Sum Sq** | **d.f** | **F** | **P** | **Partial η2** | **90% CI** |
| --- | --- | --- | --- | --- | --- | --- |
| School year | 8.575 | 1 | 4.173 | 0.041 | 0.006 | [0.000 to 0.018] |
| School timing | 40.475 | 2 | 9.848 | <0.0001 | 0.073 | [0.026 to 0.126] |
| Type of day | 482.981 | 1 | 235.014 | <0.0001 | 0.238 | [0.196 to 0.280] |
| School year:School timing | 2.612 | 2 | 0.636 | 0.530 | 0.002 | [0.000 to 0.008] |
| School year:Type of day | 10.028 | 1 | 4.880 | 0.028 | 0.006 | [0.000 to 0.019] |
| School timing:Type of day | 136.096 | 2 | 33.112 | <0.0001 | 0.081 | [0.051 to 0.112] |
| School year:School timing:Type of day | 5.643 | 2 | 1.373 | 0.254 | 0.004 | [0.000 to 0.012] |

**Supplementary Table 12. Summary results for Total Sleep Duration model (tSD, Supp. Figure 5).**

|  |  | **Beta** | **95% CI** | **t-value** | **p-value** |
| --- | --- | --- | --- | --- | --- |
| 0 | Intercept | 9.569 | [9.239 to 9.899] | 56.574 | <0.0001 |
| 1 | 5th year | 0.153 | [-0.266 to 0.572] | 0.711 | 0.477 |
| 2 | Afternoon | 0.258 | [-0.206 to 0.722] | 1.086 | 0.278 |
| 3 | Evening | -0.386 | [-0.876 to 0.103] | -1.539 | 0.124 |
| 4 | Weekdays | -2.080 | [-2.499 to -1.661] | -9.680 | <0.0001 |
| 5 | 5th year:Afternoon | -0.484 | [-1.074 to 0.105] | -1.603 | 0.109 |
| 6 | 5th year:Evening | 0.071 | [-0.551 to 0.693] | 0.223 | 0.824 |
| 7 | 5th year:Weekdays | -0.593 | [-1.186 to -0.000] | -1.951 | 0.051 |
| 8 | Afternoon:Weekdays | 0.900 | [0.310 to 1.490] | 2.978 | 0.003 |
| 9 | Evening:Weekdays | 1.785 | [1.162 to 2.407] | 5.595 | <0.0001 |
| 10 | 5th year:Afternoon:Weekdays | 0.609 | [-0.225 to 1.443] | 1.425 | 0.155 |
| 11 | 5th year:Evening:Weekdays | -0.273 | [-0.907 to 0.853] | -0.061 | 0.952 |

**Supplementary Table 13. Post-hoc comparisons for Total Sleep duration (tSD, Supp. Figure 5). Post-hoc pairwise [(1) – (2)] comparisons were significant at p<.0038 (p<.05, Bonferroni corrected).**

| **Day of the week** | **School timing (1) (2)** | | **t-value** | **df** | **p value** | **Cohen's d** | **95% CI** |
| --- | --- | --- | --- | --- | --- | --- | --- |
| Free days | Morning | Afternoon | -0.088 | 555 | 1 | -0.011 | [-0.263 to 0.240] |
| Free days | Morning | Evening | 1.810 | 555 | 0.921 | 0.245 | [-0.021 to 0.511] |
| Free days | Afternoon | Evening | 1.903 | 555 | 0.749 | 0.256 | [-0.009 to 0.521] |
| Weekdays | Morning | Afternoon | -6.645 | 555 | <0.0001 | -0.852 | [-1.106 to -0.597] |
| Weekdays | Morning | Evening | -7.325 | 555 | <0.0001 | -0.991 | [-1.260 to -0.721] |
| Weekdays | Afternoon | Evening | -1.033 | 555 | 1 | -0.139 | [-0.403 to 0.125] |
|  |  |  |  |  |  |  |  |
| **Day of the week** | **Age group (1) (2)** | |  |  |  |  |  |
| Free days | 1st year | 5th year | -0.118 | 768 | 1 | -0.011 | [-0.185 to 0.164] |
| Weekdays | 1st year | 5th year | 3.006 | 768 | 0.036 | 0.268 | [0.093 to 0.443] |
|  |  |  |  |  |  |  |  |
| **School  Timing** | **Day of the week (1) (2)** | |  |  |  |  |  |
| Morning | Free days | Weekdays | 15.641 | 768 | <0.0001 | 1.658 | [1.437 to 1.878] |
| Afternoon | Free days | Weekdays | 7.800 | 768 | <0.0001 | 0.818 | [0.609 to 1.027] |
| Evening | Free days | Weekdays | 3.635 | 768 | 0.004 | 0.423 | [0.193 to 0.652] |
|  |  |  |  |  |  |  |  |
| **Age group** | **Day of the week (1) (2)** | |  |  |  |  |  |
| 1st year | Free days | Weekdays | 9.278 | 768 | <0.0001 | 0.827 | [0.648 to 1.006] |
| 5th year | Free days | Weekdays | 12.402 | 768 | <0.0001 | 1.105 | [0.923 to 1.287] |

**Supplementary Table 14. ANOVA results for developmental change in Chronotype (MSFsc 5th year - MSFsc 1st year, Figure 2).**

|  | **Sum Sq** | **d.f** | **F** | **P** | **Partial η2** | **90% CI** |
| --- | --- | --- | --- | --- | --- | --- |
| School timing | 59.935 | 2 | 19.678 | <0.0001 | 0.135 | [0.073-0.198] |
| baseline chronotype | 244.188 | 1 | 160.343 | <0.0001 | 0.388 | [0.314-0.455] |
| School timing:baseline chronotype | 6.549 | 2 | 2.150 | 0.1186 | 0.017 | [0.000-0.047] |

**Supplementary Table 15. Summary results for developmental change in Chronotype (MSFsc 5th year - MSFsc 1^st^ year, Figure 2).**

|  |  | **Beta** | **t-value** | **p-value** |
| --- | --- | --- | --- | --- |
| 0 | Intercept | 4.068 | 7.619 | <0.0001 |
| 1 | Afternoon | 1.991 | 2.497 | 0.013 |
| 2 | Evening | 0.344 | 0.392 | 0.696 |
| 3 | Baseline chronotype | -0.711 | -7.909 | <0.0001 |
| 4 | Afternoon:baseline chronotype | -0.112 | -0.861 | 0.390 |
| 5 | Evening:baseline chronotype | 0.179 | 1.297 | 0.196 |

**Supplementary Table 16. ANOVA results for age-related change in Social jetlag (ΔSJL, SJL 5th year - SJL 1st year, Figure 3a).**

|  | **Sum Sq** | **d.f** | **F** | **P** | **Partial η2** | **90% CI** |
| --- | --- | --- | --- | --- | --- | --- |
| School timing | 8.835 | 2 | 4.493 | 0.012 | 0.034 | [0.004 to 0.075] |
| ΔChronotype | 231.825 | 1 | 235.795 | <0.0001 | 0.482 | [0.413 to 0.543] |
| School timing:ΔChronotype | 13.805 | 2 | 7.021 | 0.001 | 0.053 | [0.014 to 0.100] |

**Supplementary Table 17. Summary results for age-related change in Social jetlag (ΔSJL, SJL 5th year - SJL 1st year, Figure 3a).**

|  |  | **Beta** | **95% CI** | **t-value** | **p-value** |
| --- | --- | --- | --- | --- | --- |
| 0 | Intercept | -0.521 | [-0.757 to -0.285] | -4.351 | <0.0001 |
| 1 | Morning | 0.251 | [-0.061 to 0.563] | 1.582 | 0.115 |
| 2 | Evening | 0.267 | [-0.090 to 0.625] | 1.473 | 0.142 |
| 3 | ΔChronotype | 0.549 | [0.431 to 0.668] | 9.112 | <0.0001 |
| 4 | Morning:ΔChronotype | 0.252 | [0.073 to 0.431] | 2.767 | 0.006 |
| 5 | Evening:ΔChronotype | -0.122 | [-0.319 to 0.076] | -1.215 | 0.226 |

**Supplementary Table 18. Post-hoc comparisons for age-related change in Social jetlag (ΔSJL, SJL 5th year - SJL 1st year, Figure 3a). Post-hoc pairwise [(1) – (2)] comparisons were significant at p<.017 (p<.05, Bonferroni corrected).**

| **School timing**  **(1) (2)** | | **t-value** | **p-value** | **Cohen's d** | **95% CI** |  |
| --- | --- | --- | --- | --- | --- | --- |
| Morning | Afternoon | 2.767 | 0.017 | 0.254 | [0.073 to 0.434] | |
| Morning | Evening | 3.552 | 0.001 | 0.377 | [0.168 to 0.585] | |
| Afternoon | Evening | 1.215 | 0.446 | 0.123 | [-0.076 to 0.322] | |

**Supplementary Table 19. ANOVA results for age-related change in Sleep duration on weekdays (SDw 5th year - SDw 1st year, Figure 3b).**

|  | **Sum Sq** | **d.f** | **F** | **P** | **Partial η2** | **90% CI** |
| --- | --- | --- | --- | --- | --- | --- |
| School timing | 6.345 | 2 | 1.433 | 0.241 | 0.011 | [0.000 to 0.037] |
| ΔChronotype | 18.152 | 1 | 8.196 | 0.005 | 0.031 | [0.006 to 0.075] |
| School timing:ΔChronotype | 34.783 | 2 | 7.852 | <0.001 | 0.058 | [0.017 to 0.108] |

**Supplementary Table 20. Summary results for age-related change in Sleep duration on weekdays (SDw 5th year - SDw 1st year, Figure 3b).**

|  |  | **Beta** | **95% CI** | **t-value** | **p-value** |
| --- | --- | --- | --- | --- | --- |
| 0 | Intercept | -0.887 | [-1.194 to -0.579] | -5.682 | <0.0001 |
| 1 | Morning | -0.039 | [-0.507 to 0.430] | -0.162 | 0.871 |
| 2 | Evening | -0.080 | [-0.587 to 0.427] | -0.311 | 0.756 |
| 3 | ΔChronotype | -0.109 | [-0.310 to 0.093] | -1.062 | 0.289 |
| 4 | Morning:ΔChronotype | 0.299 | [0.031 to 0.568] | 2.193 | 0.029 |
| 5 | Evening:ΔChronotype | 0.623 | [0.313 to 0.934] | 3.950 | <0.001 |

**Supplementary Table 21. Post-hoc comparisons for age-related change in Sleep duration on weekdays (5^th^ year SDw - 1^st^ year SDw, Figure 3b). Post-hoc pairwise [(1) – (2)] comparisons were significant at p<.017 (p<.05, Bonferroni corrected).**

| **School timing**   1. **(2)** | | **t-value** | **p-value** | **Cohen's d** | **95% CI** |  |
| --- | --- | --- | --- | --- | --- | --- |
| Morning | Afternoon | -2.193 | 0.074 | -0.201 | [-0.382 to -0.021] | |
| Morning | Evening | -3.950 | <0.001 | -0.419 | [-0.628 to -0.210] | |
| Afternoon | Evening | -2.154 | 0.081 | -0.218 | [-0.417 to -0.019] | |

**Supplementary Results**

**Effect of school timing, age and baseline chronotype tertile on MSFsc**. As expected and, as also showed on Fig. 1a, chronotype depends on age (F_1,250_=59.117, P<0.0001, partial η2=0.191, 90% CI = 0.124-0.262), school timing (F_1,250_=63.008, P<0.0001, partial η2=0.335, 90% CI = 0.258-0.404) and the interaction between school timing and school year (F_2,250_=16.343, P<0.0001, partial η2=0.116, 90% CI = 0.058-0.177). Chronotype depends on chronotype tertile in 1st year (F_1,250_=145.940, P<0.0001, partial η2=0.539, 90% CI = 0.473-0.594), which is trivial. Importantly, chronotype is also modulated by the interaction between Chronotype tertile in 1st year and age (F_2,250_=46.072, P<0.0001, partial η2=0.269, 90% CI = 0.193-0.339) (Fig. 2a; Supp. Fig. 6), indicating that the trajectory (magnitude and/or direction) of the ΔChronotype depends on the initial starting point of adolescents’ chronotype. Independently on school timing, chronotype significantly changed with age in all tertiles, but the direction and the magnitude of the change differ. Chronotype became later when adolescents got older for both earlier (1.53h; 1^st^ vs. 5^th^ year: t_250_=-10.454, P<0.0001) and intermediate tertiles (0.83h; 1st vs. 5th year: t250=-5.591, P<0.0001). In the later tertile, in contrast, chronotype becomes 0.41h earlier with age (1^st^ vs. 5^th^ year: t_250_=2.868, p=0.014). The effect of age on chronotype did not depend on the interaction between 1st year tertile and school timing (F_4,250_=0.571, P=0.68, partial η2=0.009, 90% CI = 0.000-0.021): both factors independently modulated age-related changes on chronotype. Overall, the change on chronotype from 1^st^ to 5^th^ year for the earlier tertile is the largest; and, on the later tertile, the change is smaller or even negative (for all school timings)

**Supplementary Discussion**

In this study, we evaluated the same adolescents at two different time points during their development. Thus, we were able to assess whether ΔChronotype is associated with age-related changes in SJL and SDw.

Regarding SJL, we found that large chronotype delays are associated with an increase in SJL and that this association is progressively weaker from morning to evening school timings. This result was expected: sleep timing on free days (i.e. MSFsc) is delayed with age but during weekdays, this delay is limited by school schedules, leading to an increase in SJL. If school timing is early for students’ chronotype^1^, SDw is expected to be shorter when MSFsc is delayed (i.e. students would go to bed later but they would still have to wake up at the same time). However, we only observed a non-significant tendency in that direction in the morning: a 1h-delay in chronotype from 1^st^ to 5^th^ year was associated with a shortening of only 7min in SDw **(Fig. 3b)**. Interestingly and in contrast, a delay in chronotype was associated with an increase in SDw in both afternoon and evening school timings, with a steeper association for the latter. This result was not expected, but it is not surprising. While in the morning, those adolescents with later chronotypes were expected to sleep shorter due to later bedtimes (because of later internal time) but similar wake-up times (because of the early school start time), evening-attending adolescents with the earliest chronotypes might be delaying their bedtimes due to late school end-time, but waking-up earlier due to their internal timing. Consistently, those evening-attending students who experienced larger delays from 1^st^ to 5^th^ year, will probably have a better alignment between their internal timing and their school schedule in 5^th^ year, and, consequently, will have longer SDw in 5^th^ than in 1^st^ year. Thus, a better alignment between adolescents’ internal timing and school timing seems to be beneficial in terms of sleep duration not only in the morning but also in afternoon and evening school timings.

**Supplementary References**

1. Carskadon, M. A. Sleep in Adolescents: The Perfect Storm. *Pediatr. Clin. North Am.***58**, 637–647 (2011).
